# Prophylactic Evidence of MSCs-Derived Exosomes in Doxorubicin/Trastuzumab-Induced Cardiotoxicity: Beyond Mechanistic Target of NRG-1/Erb Signaling Pathway

**DOI:** 10.3390/ijms23115967

**Published:** 2022-05-25

**Authors:** Nesrine Ebrahim, Hajir A. Al Saihati, Ola Mostafa, Amira Hassouna, Sameh Abdulsamea, Eman Abd El Aziz M. El Gebaly, Nashwa Hassan Abo-Rayah, Dina Sabry, Mohamed El-Sherbiny, Abdelmonem G. Madboly, Noha Ibrahim Hussien, Raja El Hasnaoui Saadani, Hasnaa Ali Ebrahim, Omnia A. M. Badr, Nehal M. Elsherbiny, Rabab F. Salim

**Affiliations:** 1 Department of Medical Histology and Cell Biology, Faculty of Medicine, Benha University, Benha 13511, Egypt; nesrien.salem@fmed.bu.edu.eg (N.E.); ola.mostafa.moez@gmail.com (O.M.); 2 Stem Cell Unit, Faculty of Medicine, Benha University, Benha 13511, Egypt; 3 Department of Clinical Laboratory Sciences, College of Applied Medical Sciences, University of Hafr Albatin, Hafr Albatin 39524, Saudi Arabia; hajirsh@uhb.edu.sa; 4 School of Interprofessional Health Studies, Faculty of Health and Environmental Sciences, AUT University, Auckland 1010, New Zealand; amirahassona@kasralainy.edu.eg; 5 Department of Paediatrics, Faculty of Medicine, Benha University, Benha 13511, Egypt; samoha779@gmail.com; 6 Department of Clinical Pharmacology, Faculty of Medicine, Benha University, Benha 13511, Egypt; drimanpharma@gmail.com (E.A.E.A.M.E.G.); nashwa.aborayah@fmed.bu.edu.eg (N.H.A.-R.); 7 Department of Medical Biochemistry and Molecular Biology, Faculty of Medicine, Badr University in Cairo, Cairo 11562, Egypt; dinasabry@kasralainy.edu.eg; 8 Department of Medical Biochemistry and Molecular Biology, Faculty of Medicine, Cairo University, Giza 12613, Egypt; 9 Department of Basic Medical Sciences, College of Medicine, AlMaarefa University, Riyadh 11597, Saudi Arabia; msharbini@mcst.edu.sa; 10 Department of Anatomy, Mansoura Faculty of Medicine, Mansoura University, Mansoura 35516, Egypt; 11 Department of Forensic Medicine & Clinical Toxicology, Faculty of Medicine, Benha University, Benha 13511, Egypt; abdelmonem.algohari@fmed.bu.edu.eg; 12 Department of Physiology, Faculty of Medicine, Banha University, Benha 13511, Egypt; noha.ibrahim@fmed.bu.edu.eg; 13 Department of Basic Medical Sciences, College of Medicine, Princess Nourah bint Abdulrahman University, Riyadh 11671, Saudi Arabia; relhasnaui@pnu.edu.sa (R.E.H.S.); haebrahim@pnu.edu.sa (H.A.E.); 14 Department of Genetics and Genetic Engineering, Faculty of Agriculture, Benha University, Benha 13511, Egypt; omnia.badr@fagr.bu.edu.eg; 15 Department of Pharmaceutical Chemistry, Faculty of Pharmacy, University of Tabuk, Tabuk 71491, Saudi Arabia; 16 Department of Biochemistry, Faculty of Pharmacy, Mansoura University, Mansoura 35516, Egypt; 17 Department of Medical Biochemistry and Molecular Biology, Faculty of Medicine, Benha University, Benha 13511, Egypt

**Keywords:** trastuzumab, doxorubicin, stem cells, exosomes, cardiac toxicity, NRG-1, MAPK, AKT

## Abstract

Trastuzumab (Trz) is a humanized monoclonal antibody targeting epidermal growth factor receptor 2 (HER2; ErbB2). The combined administration of Trz and doxorubicin (DOX) has shown potent anti-cancer efficacy; however, this regimen may be accompanied by severe cardiac toxicity. Mesenchymal stem cells (MSCs)-derived exosomes are nanosized vesicles that play a crucial role in cell–cell communication and have shown efficacy in the treatment of various diseases. In this study, we aim to investigate the cardioprotective effects of MSCs-derived exosomes in a DOX/Trz- mediated cardiotoxicity model, and the possible mechanisms underlying these effects are elucidated. Forty-nine male rats were randomly assigned into four groups: Group I (control); Group II (Dox/Trz); Group III (protective group); and Group IV (curative group). Cardiac hemodynamic parameters, serum markers of cardiac injury, oxidative stress indices, and cardiac histopathology were investigated. Further, transcript profile of specific cardiac tissue injury markers, apoptotic markers, and fibrotic markers were analyzed using qRT-PCR, while the protein expressions of pAkt/Akt, pERK/ERK, pJNK/JNK, pJNK/JNK, and pSTAT3/STAT3 were evaluated by ELISA. Additionally, cardiac mirR-21 and miR-26a were assessed. A combined administration of DOX/Trz disrupted redox and Ca^2+^ homeostasis in cardiac tissue induced myocardial fibrosis and myofibril loss and triggered cardiac DNA damage and apoptosis. This cardiotoxicity was accompanied by decreased NRG-1 mRNA expression, HER2 protein expression, and suppressed AKT and ERK phosphorylation, while triggering JNK phosphorylation. Histological and ultra-structural examination of cardiac specimens revealed features typical of cardiac tissue injury. Moreover, a significant decline in cardiac function was observed through biochemical testing of serum cardiac markers and echocardiography. In contrast, the intraperitoneal administration of MSCs-derived exosomes alleviated cardiac injury in both protective and curative protocols; however, superior effects were observed in the protective protocol. The results of the current study indicate the ability of MSCs-derived exosomes to protect from and attenuate DOX/Trz-induced cardiotoxicity. The NRG-1/HER2, MAPK, PI3K/AKT, PJNK/JNK, and PSTAT/STAT signaling pathways play roles in mediating these effects.

## 1. Introduction

Breast cancer is one of the commonest malignancies, contributing to significant morbidity and mortality among female patients [1]. Twenty to thirty percent of breast cancer cases are associated with the over-expression of HER2/ErB2 receptors, which are related to aggressive neoplastic transformation, chemotherapy resistance, and shorter relapse time with lower overall survival rate. It has been found that HER2 over-expression also occurs in various other types of cancer, including ovary, stomach, endometrial carcinoma, uterine cervix, lung, colon, bladder, head and neck, and esophagus cancers [2].

Trastuzumab (Herceptin; Trz) is a monoclonal antibody, which has recently been utilized as a vital therapy for patients with breast cancer with HER2 receptor over-expression. Trz acts by inhibiting the HER2-mediated malignant transformation by binding to the extracellular segment of active HER2 and inhibiting its dimerization [3]. Furthermore, the therapeutic efficiency of Trz can be attributed to the inhibition of HER2 signaling through down-regulation of plasma membrane HER2 expression, enhancing its turnover and inducing endocytosis. However, it has been found that the utilization of Trz as a monotherapy agent has a deficient anti-malignant effect, necessitating its combination with other chemotherapeutic drugs, such as anthracyclines [4].

Doxorubicin (Adriamycin; DOX) is an anthracycline antibiotic, which has been broadly utilized for treating a wide range of cancers. It works by interposing into the DNA double helix, leading to inhibition of the topoisomerase II, thus preventing DNA replication [5]. The combination of Trz and DOX has shown a more efficient anti-malignant activity than Trz alone. Data have shown that this regimen improved the pathological complete response by 50%, consequently establishing itself as the typical neo-adjuvant chemotherapy against HER2-positive patients with breast cancer [6]. However, this combination is challenged by low selectivity and severe cardiotoxicity, manifesting either as acute or chronic ventricular systolic impaired function, causing heart failure in about 27% of the treated patients [7]. Therefore, ensuring a cardio-protective effect in the adjuvant Trz/DOX regimen is of ultimate clinical necessity, with potential value for patients with HER2-positive breast cancer.

In this context, several studies have been carried out to offer selective drug delivery and diminish the DOX/Trz-associated cardiotoxicity. Indeed, this cardiotoxicity has been established due to Trz-mediated specific recognition of the tumor antigen HER2. Nevertheless, this line of treatment is blocked by the ‘binding site barrier’ effect, leading to incomplete tumor infiltration.

Over the past decade, mesenchymal stem cells (MSCs) have gained increasing interest in research as a unique means for protective and regenerative therapy guarding against cardiac damage [8]. MSCs possess the capability to differentiate into many different lineages. The therapeutic effects of MSCs are mostly due to their paracrine effect through exosomes [9]. These exosomes, considered as extracellular nano-vesicles released by the budding of the cellular endosomal membranes, are vitally involved in cell–cell communication. Exosomes convey lipids, proteins, miRNA, and mRNA cargo, which can be functionally transported to affect the target cells. Stem-cell-derived exosomes have been considered as striking candidates for cell-based therapies and have shown great efficacy in the treatment of numerous diseases [10].

The cardiotoxic effect of DOX is mainly due to the triggering of oxidative stress and inflammation in the myocardium and vasculature, leading to myocardial apoptosis, fibrosis, and heart failure [11]. DOX activates redox-sensitive transcription factors—mainly nuclear factor κB (NFκB)—which, in turn, provokes the expression of pro-inflammatory cytokines. These up-regulated inflammatory cytokines mediate myocardial fibrosis, myocarditis, and heart failure. Moreover, DOX-induced oxidative stress activates MAPK, which prompts the expression of various pro-apoptotic proteins [12].

DOX-induced stress on cardiomyocytes can be alleviated by the HER2/neuregulin-1 signaling pathway. Neuregulin-1 (NRG-1), a member of the neuregulin growth factor family, is involved in many cellular processes. The activity of NRG-1 is mediated through paracrine and juxtacrine signaling by receptor tyrosine kinases termed erythroblastic leukemia viral oncogene homologs (ErbBs). NRG-1 binds to the membrane receptor tyrosine kinase ErbB4. Ligand/receptor binding leads to homodimerization of ErbB4 or its heterodimerization with ErbB2, followed by consequent transphosphorylation of the ErbB cytoplasmic signaling tails, triggering intracellular activation of downstream pro-survival signals, including Src/focal adhesion kinase, MAPK/ERK, phosphatidylinositide 3-kinase (PI3K)/Akt, and nitric oxide synthase (NOS) [13]. Of note, an over-expression of ErbB2/HER2 reduces the basal levels of reactive oxygen species (ROS), up-regulates antioxidant enzymes, and protects against DOX-induced cardiac injury [14]. Dilated cardiomyopathy was consistently developed in cardiac-restricted ErbB2 knockout mice [15]. Therefore, the inhibition of HER2 by Trz exacerbates DOX-induced stress on the myocardium [16].

In the present study, we aimed to investigate the therapeutic value of adipose tissue-MSCs (ADMSCs)-derived exosomes, either in protective or curative protocols, against Trz/DOX-induced cardiotoxicity, unraveling their impact on the NRG-1, MAPK, STAT, and PI3K/AKT signaling pathways.

## 2. Results

### 2.1. Characterization and Identification of Exosomes

**Figure 1:**
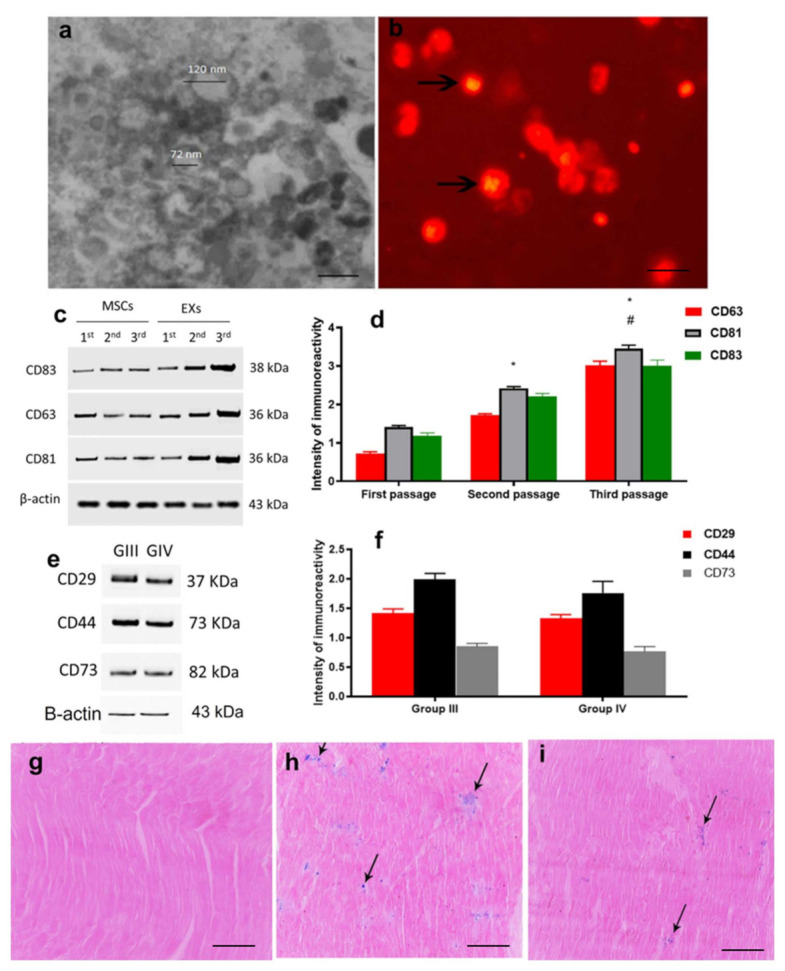
(**a**) TEM of exosomes showing spheroid double-membrane-bound morphology (arrows) with a diameter of 40–100 nm; (**b**) Exosomes were also detected in cardiac tissues by PKH26; (**c**) Western blot for exosome characterization; (**d**) Histogram of exosome characterization for CD81, CD63, CD83 indicated significant differences (*p* ˂ 0.05) *, significant compared to the first passage at *p* ˂ 0.05; #, significant compared to the second passage at *p* ˂ 0.05. Data are shown as mean ± SEM; (**e**) Western blot for exosome localization in cardiac tissues; (**f**) Histogram of exosome localization in groups III and IV for CD29, CD44, and CD73. Data are shown as mean ± SEM. Prussian blue staining of cardiac tissues proved the absence of blue staining in un-injected animals (**g**) and iron oxide particles stained blue in the myocardium of exosome-injected animals in the protective (**h**) and curative (**i**) groups.

Transmission electron microscopy examination of purified exosomes demonstrated their characteristic spheroid double-membrane-bound morphology with a diameter of 40–120 nm (Figure 1a). Further, exosomes labeled with PKH26 dye, a red fluorescence chromophore inserted into the lipid bilayers of exosomes, were incorporated in cardiac tissue, which became visible under fluorescent microscopy (Figure 1b). The isolated exosomes had specific membrane markers, such as CD63, CD81, and CD83, which were scanned versus β actin in different passages by Western blot analysis (Figure 1c,d). The detected levels of CD63, CD81, and CD83 showed a gradual increase in isolated exosomes while moving from one passage to another, as the quantitative expression of CD63, CD81, and CD83 against β actin showed a significant increase in passage 2, when compared to passage 1, as well as a significant increase in passage 3, when compared to passage 2. The localization of exosomes within the cardiac tissues in groups III and IV was confirmed by detecting CD29, CD44, and CD73 (Figure 1e,f). The exosomes labeled with iron oxide injected intraperitoneally were observed in the cardiac tissue under a light microscope after staining with Prussian blue, in comparison to cardiac tissue from un-injected animals (Figure 1g–i).

### 2.2. ADMSCs-Derived Exosomes Restored DOX/Trz-Induced Alteration in Cardiac Function and Serum Cardiotoxicity Indices

**Figure 2:**
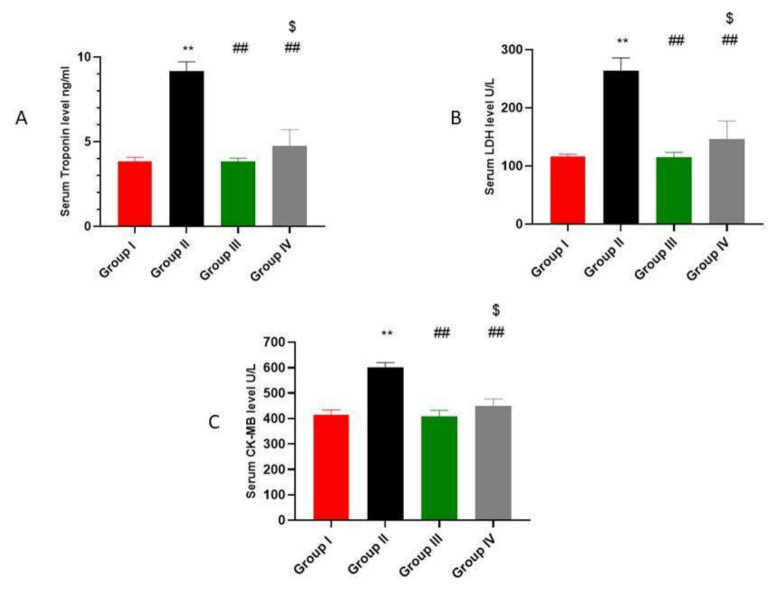
Effect of ADMSCs-derived exosomes on DOX/Trz-induced cardiac dysfunction: (**A**) Serum troponin level (ng/mL); (**B**) Serum lactate dehydrogenase (LDH) U/L; (**C**) Serum creatine kinase (CK-MB) U/L. Results are expressed as mean ± SEM. **, significant compared to Group I at *p* ˂ 0.01; ##, significant compared to Group II at *p* ˂ 0.01; $, significant compared to Group III at *p* ˂ 0.05 (*n* = 6).

To ensure successful induction of the cardiotoxicity model, we assessed the biomarkers of cardiac function. As shown in Figure 2A–C, DOX/Trz administration forced significant increases in serum troponin, LDH, and CK-MB, by 2.83-, 2.27-, and 1.44-fold, respectively, compared to the control group. The administration of ADMSCs-derived exosomes resulted in a significant decrease in serum troponin, LDH, and CK-MB, by about 58%, 56%, and 32% in the protective protocol and 48%, 44%, and 25% in the curative protocol, respectively, when compared to the DOX/Trz group.

These results were further confirmed by echocardiography. To ensure successful induction of the cardiotoxicity model, we assessed cardiac hemodynamic parameters, such as LVSD, LVDD, FS, and EF, as indices of cardiac contraction and relaxation. DOX/Trz administration caused a significant decrease (*p* < 0.05) in FS% and EF%, and a significant increase (*p* < 0.05) in LVSD and LVDD, when compared to the control group. The results indicated that the cardiotoxicity of DOX/Trz seriously impaired the rat’s myocardial contractile function. However, significant increases in FS% and EF% (*p* < 0.05), as well as decreases in LVSD and LVDD (*p* < 0.05), were detected in the prophylactic and curative groups after treatment with ADMSCs-derived exosomes when compared to the DOX/Trz group. Moreover, the protective group showed significantly increased FS% and EF% (*p* < 0.05) and significantly decreased LVSD (*p* < 0.05) when compared to the curative group, which indicated that the protective group had more favorable effects on the cardiac dysfunction in the cardiotoxic group by reverting all the indices to close to normal levels (Table 1).

### 2.3. ADMSCs-Derived Exosomes Ameliorated DOX/Trz-Induced Cardiac Tissue Injury

**Figure 3:**
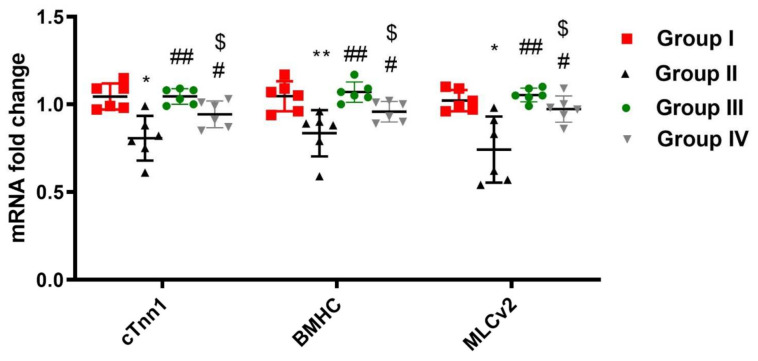
Effect of ADMSCs-derived exosomes on DOX/Trz-induced myofibril loss. Cardiac mRNA levels of cTnn1, BMHC, and MLCv2. Data expressed as median (maximum, minimum). *, significant compared to Group I at *p* ˂ 0.05; **, significant compared to Group I at *p* ˂ 0.01; #, significant compared to Group II at *p* ˂ 0.05; ##, significant compared to Group II at *p* ˂ 0.01; $, significant compared to Group III at *p* ˂ 0.05; *n* = 6.

DOX/Trz-induced cardiac toxicity was associated with myofibril loss. This was manifested as a marked reduction in mRNA expression of cTnn1, BMHC, and MLCv2 in cardiac tissue by about 23%, 20%, and 27%, respectively, when compared with normal controls. Treatment with ADMSCs-derived exosomes significantly restored mRNA expression of cardiac cTnn1, BMHC, and MLCv2 by 1.29-, 1.28-, and 1.42-fold in the protective protocol, and by 1.16-, 1.14-, and 1.3-fold in the curative protocol, respectively, when compared to the DOX/Trz group (Figure 3).

### 2.4. ADMSCs-Derived Exosomes Attenuated DOX/Trz-Induced Cardiac Oxidative Stress

**Figure 4:**
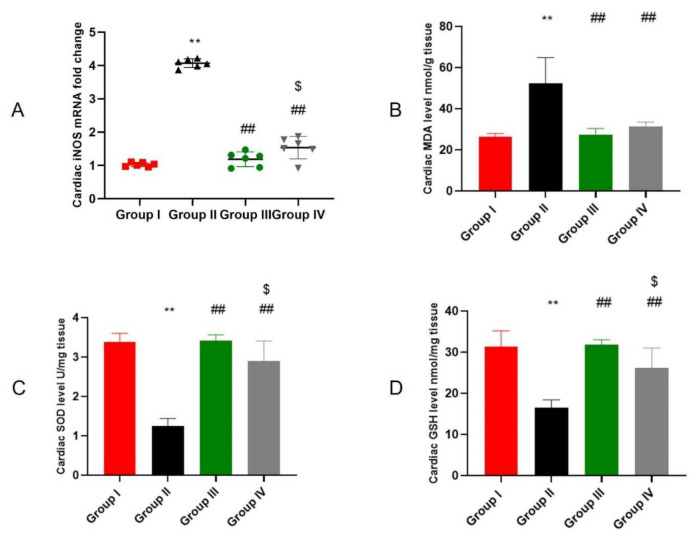
Effect of ADMSCs-derived exosomes on DOX/Trz-induced cardiac oxidative stress: (**A**) Cardiac inducible nitric oxide synthase (iNOS). Data are expressed as median (maximum and minimum); (**B**) Cardiac level of malondialdehyde (MDA); (**C**) Cardiac level of superoxide dismutase (SOD); and (**D**) Cardiac level of reduced glutathione (GSH). Results are expressed as mean ± SEM. **, significant compared to Group I at *p* ˂ 0.01; ##, significant compared to Group II at *p* ˂ 0.01; $, significant compared to Group III at *p* < 0.05; *n* = 6.

Oxidative stress is a hallmark of DOX/Trz-induced cardiotoxicity. As shown in Figure 4A, the iNOS mRNA level was significantly increased by 3.9-fold in cardiac tissues of the DOX/Trz group compared to normal controls. However, ADMSCs-derived exosome treatment markedly reduced iNOS mRNA levels; namely, by 71% in the protective protocol and by 62% in the curative protocol when compared to the DOX/Trz group. Additionally, the DOX/Trz treatment group showed marked lipid peroxidation, as demonstrated by a significant increase in cardiac MDA levels, by 1.99-fold compared to the control group. Treatment with ADMSCs-derived exosomes significantly suppressed cardiac MDA levels by 48% in the protective protocol and by 40% in the curative protocol when compared to the DOX/Trz group (Figure 4B). On the other hand, the DOX/Trz treatment group presented significant down-regulation of the cardiac levels of the antioxidant elements GSH and SOD (by 63% and 47%, respectively) when compared to the control group. However, ADMSCs-derived exosome treatment groups showed significant restoration of cardiac GSH and SOD, by 2.7- and 1.9-fold in the protective protocol and 2.3- and 1.5-fold in the curative protocol, respectively, when compared to the DOX/Trz group (Figure 4C,D).

### 2.5. ADMSCs-Derived Exosomes Ameliorated DOX/Trz-Induced DNA Damage and Apoptosis in Cardiac Tissues

**Figure 5:**
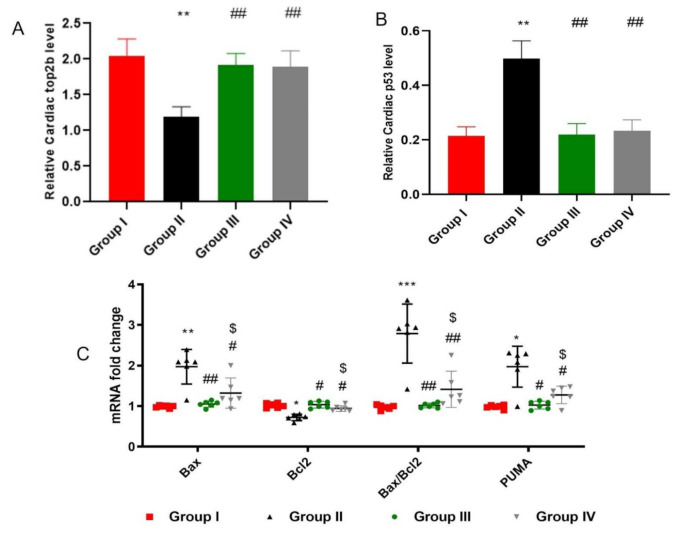
Effect of ADMSCs-derived exosomes on DOX/Trz-induced cardiac DNA damage and apoptosis: (**A**) Topoisomerase 2 beta (Top2b) and (**B**) p53 concentration by ELISA. Results are expressed as mean ± SEM; and (**C**) mRNA levels of cardiac Bax, Bcl-2, Bax/Bcl2 ratio, and PUMA. Data are expressed as median (maximum, minimum). *, significant compared to Group I at *p* ˂ 0.05; **, significant compared to Group I at *p* ˂ 0.01; ***, significant compared to Group I at *p* ˂ 0.001; #, significant compared to Group II at *p* ˂ 0.05; ##, significant compared to Group II at *p* ˂ 0.01; $, significant compared to Group III at *p* ˂ 0.05; *n* = 6.

DOX/Trz treatment down-regulated cardiac TOP2B, which is a major intracellular target that mediates DOX-induced cardiotoxicity. This, in turn, induced DNA damage and triggered apoptosis. As shown in Figure 5A,B, the DOX/Trz treatment group consistently demonstrated a marked decrease in TOP2B level in cardiac tissue (by 83.75%) compared to the control group. On the other hand, treatment with ADMSCs-derived exosome significantly restored cardiac TOP2B levels (6.11-fold in the protective protocol and 4.88-fold in the curative protocol) when compared to the DOX/Trz group. Apoptosis was evident in DOX/Trz treatment group, as revealed by significant increases in the mRNA levels of BAX and PUMA, the BAX/Bcl2 ratio, and p53 protein level, by 1.99-, 2-, 2.86-, and 4.25-fold, respectively, accompanied by a significant decrease in the Bcl2 mRNA level (by 29%) when compared to the control group. In contrast, ADMSCs-derived exosome treatment down-regulated the aforementioned apoptotic markers by 47%, 48%, 64%, and 70.8% in the protective protocol and by 33%, 35%, 49%, and 76.5% in the curative protocol, respectively, and up-regulated anti-apoptotic Bcl2 by 1.4-fold in the protective protocol and 1.3-fold in the curative protocol when compared to that in the DOX/Trz group (Figure 5A,C).

### 2.6. ADMSCs-Derived Exosomes Attenuated DOX/Trz-Induced Fibrosis in Cardiac Tissues

**Figure 6:**
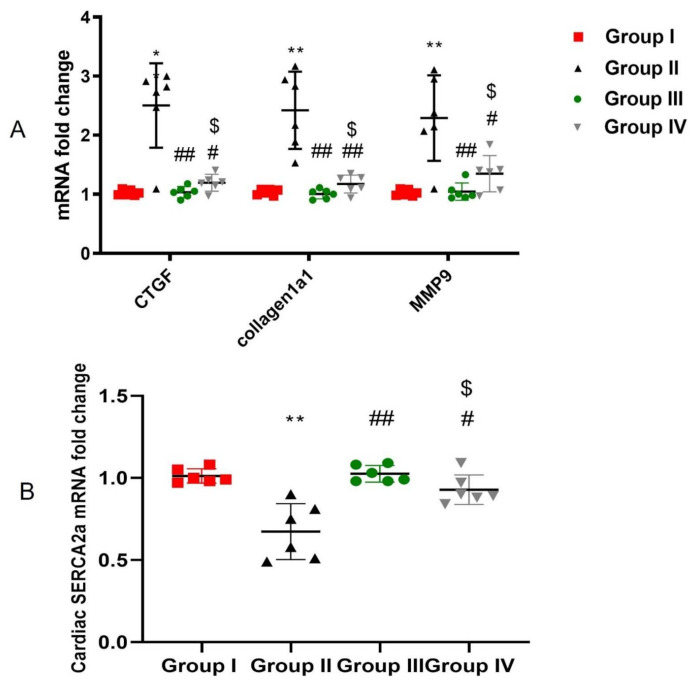
Effect of ADMSCs-derived exosomes on DOX/Trz-induced cardiac fibrosis and Ca^2+^ homeostasis: (**A**) mRNA levels of cardiac connective tissue growth factor (CTGF), collagen1a1, and Matrix metallopeptidase 9 (MMP9); and (**B**) mRNA levels of cardiac SERCA2a. Data are expressed as median (maximum, minimum). *, significant compared to Group I at *p* ˂ 0.05; **, significant compared to Group I at *p* ˂ 0.01; #, significant compared to Group II at *p* ˂ 0.05; ##, significant compared to Group II at *p* ˂ 0.01; $, significant compared to Group III at *p* ˂ 0.05; *n* = 6.

DOX/Trz cardiac toxicity is accompanied by marked fibrosis and collagen deposition. The administration of DOX/Trz resulted in increased mRNA levels of the fibrotic markers CTGF, Collagen1a1, and MMP9, by 2.44-, 2.33-, and 2.24-fold in the cardiac tissue of treated rats compared to that of normal controls. On the other hand, ADMSCs-derived exosome treatment significantly decreased the mRNA levels of these cardiac fibrotic markers by 59%, 59%, and 54% in the protective protocol and 52%, 52%, and 41% in the curative protocol, respectively, compared to those in the DOX/Trz group (Figure 6A).

### 2.7. ADMSCs-Derived Exosomes Improved Cardiac Ca^2+^ Homeostasis in DOX/Trz-Treated Rats

DOX/Trz cardiac toxicity is also associated with the dysregulation of intracellular calcium homeostasis, as demonstrated by decreased expression levels of SERCA2a mRNA in rat cardiac tissue (by 33%) compared to untreated normal controls. However, treatment with ADMSCs-derived exosomes restored cardiac SERCA2a mRNA levels, which increased by 1.5-fold in the protective protocol and by 1.3-fold in the curative protocol compared to the DOX/Trz group (Figure 6B).

### 2.8. ADMSCs-Derived Exosomes Restored NRG-1/HER2 Signaling in Cardiac Tissue of DOX/Trz-Treated Rats

**Figure 7:**
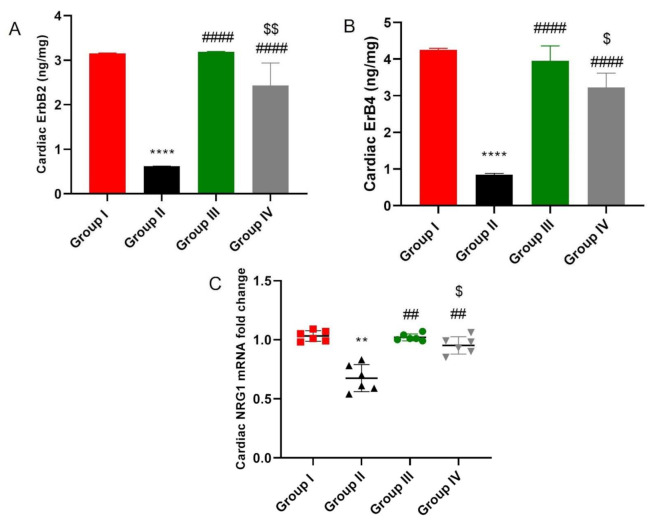
Effect of ADMSCs-derived exosomes on NRG-1/HER2 signaling in cardiac tissue of DOX/Trz-treated rats: (**A**,**B**) HER2 and ERB4 concentration determined by ELISA. Results are expressed as mean ± SEM; and (**C**) mRNA levels of cardiac NRG-1. Data are expressed as median (maximum, minimum). **, significant compared to Group I at *p* ˂ 0.01; ****, significant compared to Group I at *p* ˂ 0.0001; ##, significant compared to Group II at *p* ˂ 0.01; ####, significant compared to Group II at *p* ˂ 0.0001; $, significant compared to Group III at *p* ˂ 0.05; *n* = 6.

As shown in Figure 7, DOX/Trz treatment resulted in significant down-regulation of cardiac NRG-1/HER2 signaling, as indicated by the significant suppression of cardiac NRG-1 mRNA and HER2 and ERB4 proteins by 35%, 80.4%, and 80.1%, respectively, compared to the normal control group. Nevertheless, the administration of ADMSCs-derived exosomes markedly restored NRG-1 mRNA and HRE2 and ERB4 proteins by 1.5-, 5.16-, and 4.6-fold in the protective protocol and 1.4-, 3.9-, and 3.8-fold in the curative protocol, respectively, compared to the DOX/Trz group.

### 2.9. ADMSCs-Derived Exosomes Increased Expression of miR-21 and miR-26a Restraining PTEN in Cardiac Tissue of DOX/Trz-Treated Rats

**Figure 8:**
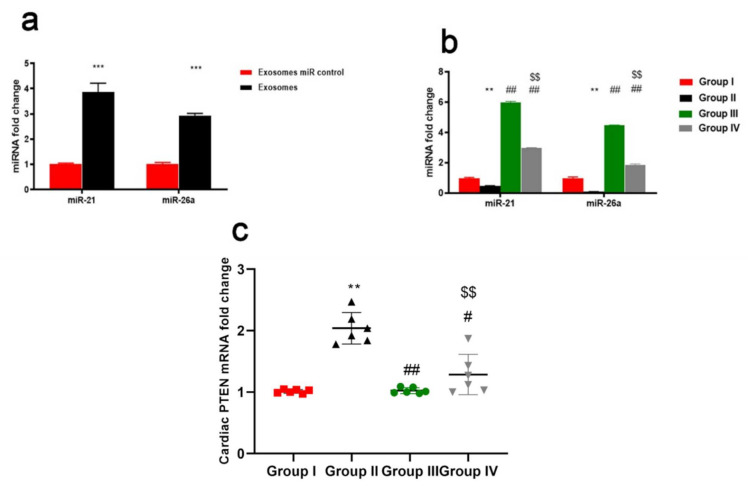
Effect of ADMSCs-derived exosomes on the expression of both miR-21 and miR-26a and PTEN in cardiac tissue of DOX/Trz-treated rats; (**a**) Quantitative analysis for relative expression of exosomal miR-21 and miR-26a; (**b**) Quantitative analysis for relative expression of miR-21 and miR-26a. Results are expressed as mean ± SEM; and (**c**) mRNA levels of cardiac PTEN. Data are expressed as median (maximum, minimum). **, significant compared to Group I at *p* ˂ 0.01; ***, significant compared to Group I at *p* ˂ 0.001; #, significant compared to Group II at *p* ˂ 0.05; ##, significant compared to Group II at *p* ˂ 0.01; $$, significant compared to Group III at *p* ˂ 0.01; *n* = 6.

qRT-PCR revealed that the expression of both miR-21 and miR-26a was significantly increased in exosomes relative to the control group (Figure 8a). The cardiac expression of both miR-21 and miR-26a was significantly down-regulated in DOX/Trz-treated rats when compared with the control group (*p* < 0.01). However, Groups III and IV both displayed a significant up-regulation of both miR-21 and miR-26a when compared with the DOX/Trz-treated group (*p* < 0.01). Significant up-regulation of both miR-21 and miR-26a was noted for group III versus group IV (Figure 8b).

### 2.10. ADMSCs-Derived Exosomes Modulated pAKT/AKT, pERK/ERK, pJNK/JNK, and PSTAT/STAT in Cardiac Tissue of DOX/Trz-Treated Rats

**Figure 9:**
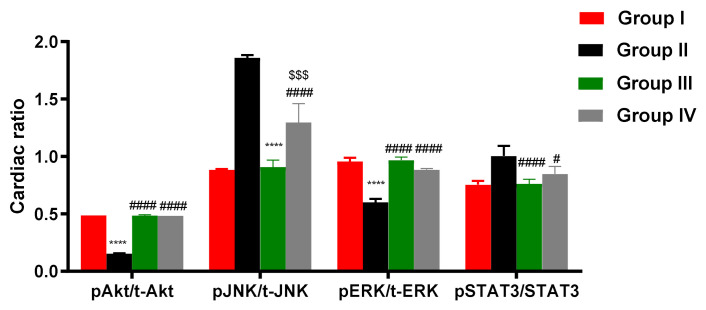
Effect of ADMSCs-derived exosomes on pAKT/AKT, pERK/ERK, pJNK/JNK, and PSTAT3/STAT3 in cardiac tissue of DOX/Trz-treated rats: (A) pAKT/AKT, pERK/ERK, and pJNK/JNK; and (B) PSTAT3/STAT3. Results are expressed as mean ± SEM. ****, significant compared to Group I at *p* ˂ 0.0001; #, significant compared to Group II at *p* ˂ 0.05; ####, significant compared to Group II at *p* ˂ 0.0001; $, significant compared to Group III at *p* ˂ 0.05; $$$, significant compared to Group III at *p* ˂ 0.001; *n* = 6.

ELISA demonstrated suppressed phosphorylation of AKT and ERK but elevated phosphorylation of JNK and STAT3 in the cardiac tissue of DOX/Trz-treated rats. In contrast, ADMSCs-derived exosome treatment groups (both protective and curative protocols) showed restored AKT and ERK phosphorylation and suppressed JNK and STAT3 phosphorylation in cardiac tissues when compared to the DOX/Trz group (Figure 9). Interestingly, these results were accompanied by a significant increase in the mRNA level of PTEN, an inhibitor of AKT signaling in the cardiac tissue of DOX/Trz-treated rats; however, ADMSCs-derived exosome treatment suppressed PTEN mRNA levels in the cardiac tissue of treated rats under both protective and curative protocols (Figure 8c).

### 2.11. ADMSCs-Derived Exosomes Restored DOX/Trz-Induced Alteration in Cardiac Muscle Histopathology

Histological examination of the different sub-groups of Group I (control group) showed similar results; therefore, the results of subgroup Ia are used to represent this group.

#### 2.11.1. Light Microscope Examination

##### H&E Staining Results

**Figure 10:**
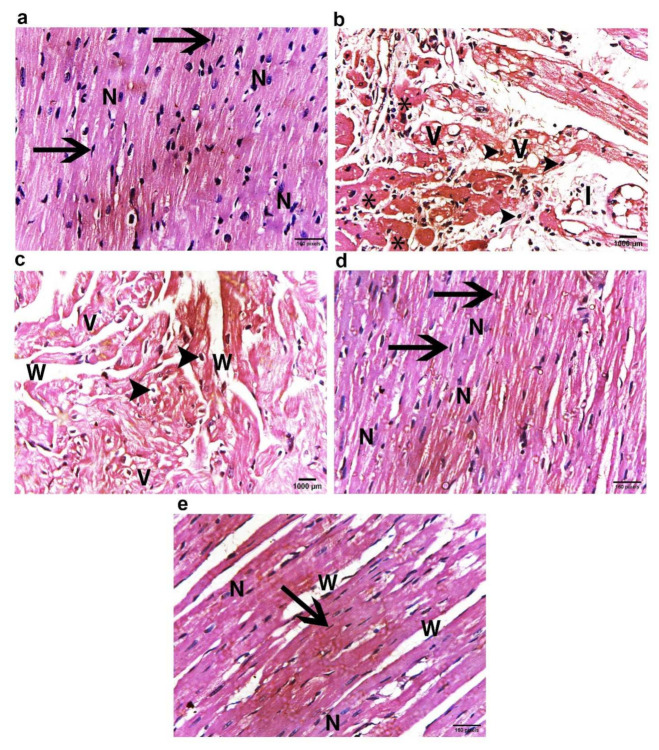
Representative photomicrographs of hematoxylin- and eosin-stained sections from the left ventricular myocardium in the different experimental groups: (**a**) Group I (control group) showed anastomosing and branching cardiac muscle fibers. The cardiomyocytes displayed central vesicular oval nuclei (N) and acidophilic cytoplasm. The flat dark nuclei (arrow) of the endomysium connective tissue fibroblasts are shown; (**b**) Group IIa (DOX/Trz group) showed disrupted and separated myofibers with intracytoplasmic vacuolization (V), hyaline degeneration, and Zenker’s necrosis (*) and pyknosis (arrowhead) of many cardiomyocyte nuclei. Notice the accumulation of inflammatory cells (I) among the myofibers; (**c**) Group IIb (recovery group) showed separated, thinly stretched myofibers with the absence of striation (W), cytoplasmic vacuolization (V), and pyknosis (arrowhead) of numerous cardiomyocyte nuclei; (**d**) Group III (prophylactic group) showed a nearly normal histological architecture, with acidophilic cytoplasm and vesicular, oval centrally located nuclei (N). Notice the regularly arranged muscle fibers with narrow spacing; and (**e**) Group IV (curative group) showed slightly disorganized cardiac muscle fibers with slight narrow spacing between the myofibers (W); *n* = 6.

The histopathological changes in the left ventricular myocardium of all study groups are shown in Figure 10. The control group displayed the normal histological architecture of the myocardium. The myocytes were cylinder shaped, branching with acidophilic sarcoplasm and single central vesicular oval nucleus. The blood capillaries were in the intercellular spaces (Figure 10a). On the other hand, group IIa (Dox/Trz group) showed significant alterations in the myocardium, in the form of interstitial edema, intracytoplasmic vacuolization, hyaline degeneration, and Zenker’s necrosis (coagulative necrosis) with mononuclear inflammatory cell infiltration. The nuclei were peripherally positioned and became pyknotic. Moreover, myofibril disorganization was observed, with marked variation in myofibril diameter and fine chicken-wire-like interstitial fibrosis (Figure 10b). Furthermore, group IIb (recovery group) showed a similar picture to group IIa but with less inflammatory cell infiltration (Figure 10c). Meanwhile, in exosome-treated groups (groups III and IV), the myocardial damage improved dramatically after exosome injections when compared to the DOX/Trz group, as the myocardium showed decreased necrosis, intracytoplasmic vacuolization, and maintained a normal structure and morphology of the myocardium, with no inflammatory cells in group IV, reaching a near-normal structure and morphology in the prophylactic group (group III); see Figure 10 d,e.

#### 2.11.2. Masson’s Trichrome Stain for Collagen Fibers

**Figure 11:**
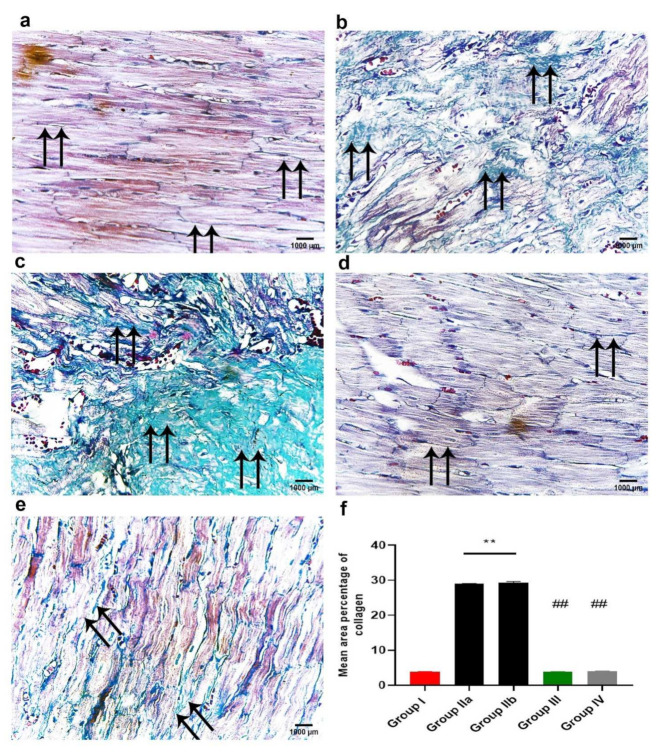
Representative photomicrographs of Masson’s trichrome-stained sections from the left ventricle of the myocardium in the different experimental groups: (**a**) Group I (control group) showed minimal collagen fibers between cardiac muscle fibers (double arrow); (**b**) Group IIa (DOX/Trz group) showed moderate collagen fiber deposition among cardiac muscle fibers (double arrow); (**c**) Group IIb (recovery group) showed marked collagen fiber accumulation between cardiac muscle fibers (double arrow); (**d**) Group III (prophylactic group) showed minimal collagen fiber deposition among cardiac muscle fibers (double arrow); (**e**) Group IV (curative group) showed few collagen fibers among cardiac muscle fibers (double arrow); and (**f**) Histogram representing the mean area percentage of collagen fibers in all experimental groups. **, significant compared to Group I at *p* ˂ 0.01; ##, significant compared to Group II at *p* ˂ 0.01; *n* = 6.

Masson’s trichrome staining was used for evaluation of collagen fibers in cardiac muscle that might be located in the extra-myocyte space. These changes are shown in Figure 11. Group I (control group) showed minimal collagen fiber deposition among the cardiac muscle fibers, as well as around the wall of capillaries (Figure 11a). However, group IIa (DOX/Trz group) showed increased collagen fiber deposition, which was more extensive in the recovery group (group IIb) compared with the control group (Figure 11b,c). Meanwhile, exosome-treated groups (groups III and IV) showed lower collagen fiber content between the cardiac myocytes, reaching a normal level in the prophylactic group (group III); see Figure 11d,e. 

#### 2.11.3. Immunohistochemistry Results

**Figure 12:**
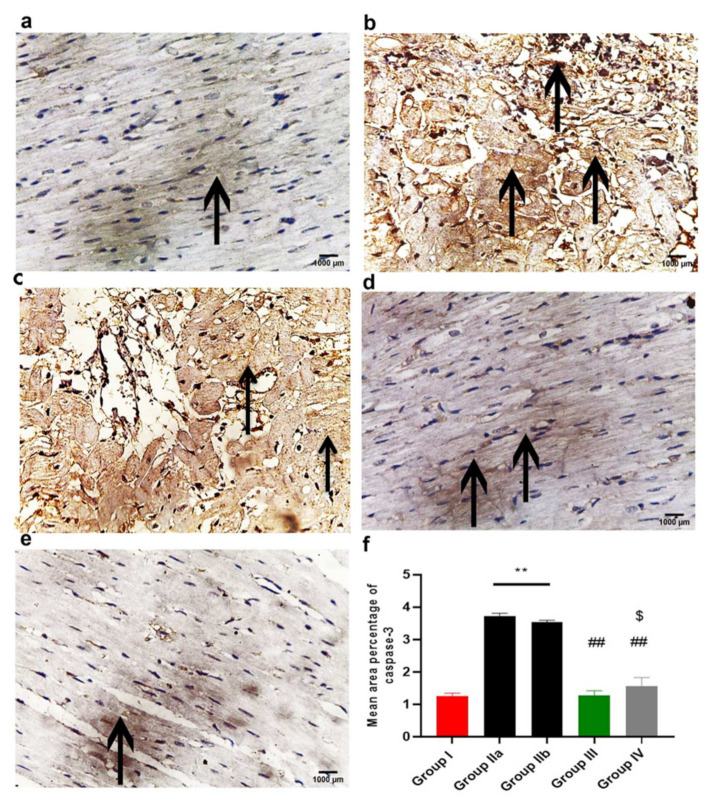
Representative photomicrographs of caspase-3 immuno-stained sections from the left ventricle of the myocardium in the different experimental groups: (**a**) Group I (control group) showed minimal cytoplasmic immuno-expression of caspase-3 in the myocardium; (**b**) Group IIa (DOX/Trz group) showed marked cytoplasmic and nuclear immuno-expression of caspase-3 in the myocardium; (**c**) Group IIb (recovery group) showed moderate cytoplasmic and nuclear immuno- expression of caspase-3 in the myocardium; (**d**) Group III (prophylactic group) showed minimal cytoplasmic immuno-expression of caspase-3 in the myocardium; (**e**) Group IV (curative group) showed low cytoplasmic immuno-expression of caspase-3 in the myocardium; and (**f**) Histogram representing the mean area percentage of caspase-3 immuno-reaction in all experimental groups. **, significant compared to Group I at *p* ˂ 0.01; ##, significant compared to Group II at *p* ˂ 0.01; $, significant compared to Group III at *p* ˂ 0.05; *n* = 6.

The immunohistochemical reaction of Caspase-3 is shown in Figure 12. The immune expression of Caspase-3 is cytoplasmic and nuclear. The control group (group I) showed very weak reactions in some cardiac muscle fibers (Figure 12a), while groups IIa and b (DOX/Trz and recovery groups) showed an increased sarcoplasmic and nuclear immuno- reaction, which was more diffuse and extensive in many cardiac muscle fibers (Figure 12b,c). Meanwhile, in the exosome-treated groups (groups III and IV), decreased immunoreaction in cardiac muscle fibers was observed, being very minimal in group III (prophylactic group); see Figure 12d,e.

**Figure 13:**
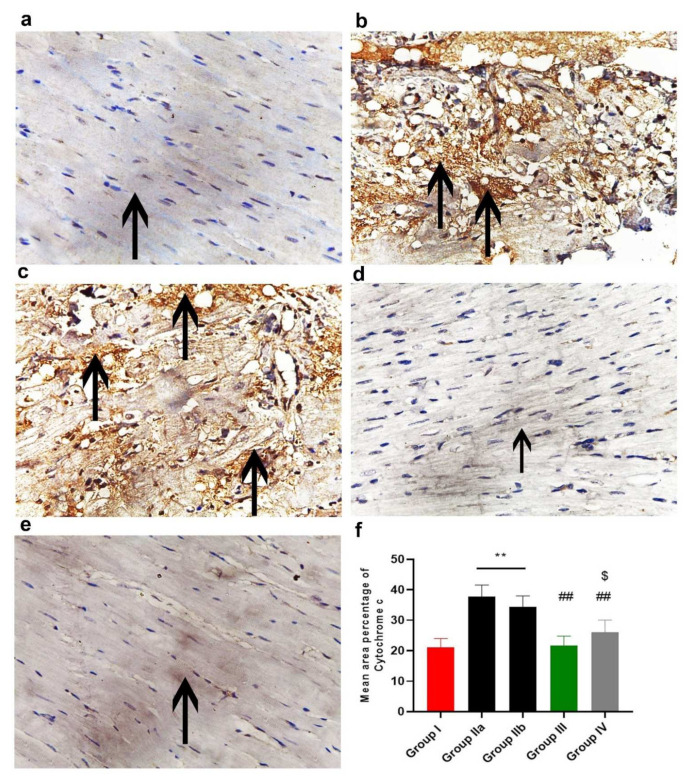
Representative photomicrographs of cytochrome C immuno-stained sections from the left ventricle of the myocardium in the different experimental groups: (**a**) Group I (control group) showed minimal cytoplasmic immune expression of cytochrome C in the myocardium; (**b**) Group IIa (DOX/Trz group) showed marked cytoplasmic immuno-expression of cytochrome C in the myocardium; (**c**) Group IIb (recovery group) showed moderate cytoplasmic immuno-expression of cytochrome C in the myocardium; (**d**) Group III (prophylactic group) showed minimal cytoplasmic immuno-expression of cytochrome C in the myocardium; (**e**) Group IV (curative group) showed few cytoplasmic immuno-expression of cytochrome C in the myocardium; and (**f**) Histogram representing the mean area percentage of cytochrome C immuno-reaction in all experimental groups. **, significant compared to Group I at *p* ˂ 0.01; ##, significant compared to Group II at *p* ˂ 0.01; $, significant compared to Group III at *p* ˂ 0.05; *n* = 6.

The immunohistochemical reaction of Cytochrome C is shown in Figure 13. The control group (group I) showed a very weak reaction in the sarcoplasm of some cardiac muscle fibers (Figure 13a). Groups IIa and b (DOX/Trz and recovery groups) showed increased sarcoplasmic immuno-reaction, which was more diffuse and extensive in many cardiac muscle fibers (Figure 13b,c). In contrast, the exosome-treated groups (groups III and IV) showed decreased immuno-reaction in cardiac muscle fibers, which was very minimal in group III (prophylactic group); see Figure 13d,e.

**Figure 14:**
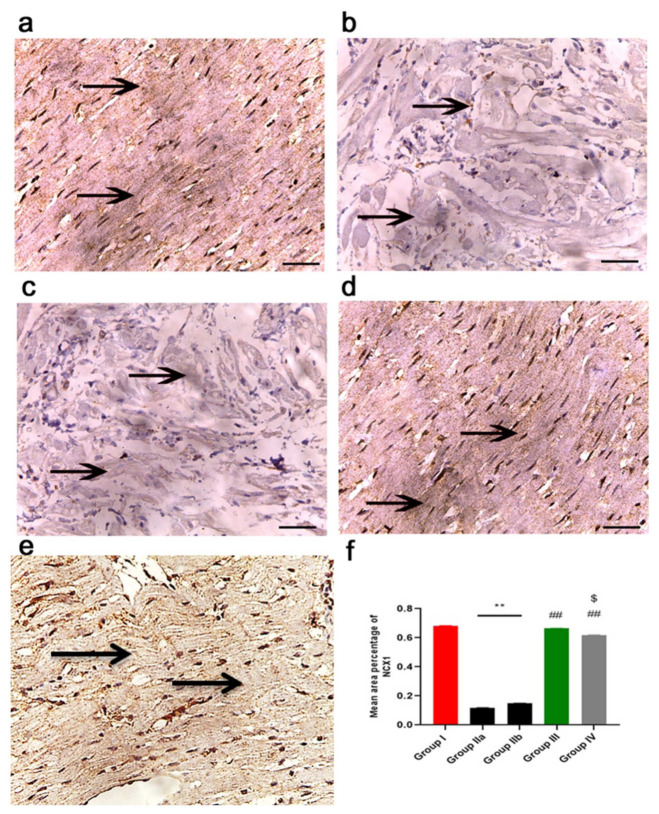
Representative photomicrographs of NCX1 immuno-stained sections from the left ventricle of the myocardium in the different experimental groups: (**a**) Group I (control group) showed strong immuno-expression of NCX1 in the myocardium; (**b**) Group IIa (DOX/Trz group) showed minimal immuno-expression of NCX1 in the myocardium; (**c**) Group IIb (recovery group) showed near-minimal immuno-expression of NCX1 in the myocardium; (**d**) Group III (prophylactic group) showed strong immuno-expression of NCX1 in the myocardium; (**e**) Group IV (curative group) showed moderate immuno-expression of NCX1 in the myocardium; and (**f**) Histogram representing the mean area percentage of NCX1 immuno-reaction in all experimental groups. **, significant compared to Group I at *p* ˂ 0.01; ##, significant compared to Group II at *p* ˂ 0.01; $, significant compared to Group III at *p* ˂ 0.05; *n* = 6.

The immunohistochemical reaction of NCX1 is shown in Figure 14. The sodium–calcium exchanger (NCX1) is a key sarcolemmal protein for the maintenance of calcium homeostasis in the heart, as it facilitates Ca^2+^ influx under specialized physiological circumstances. Staining is localized to the sarcoplasm and cell membrane. The control group (group I) showed a strong immune reaction (Figure 14a), while groups IIa and b (DOX/Trz and recovery groups) showed decreased immuno-reaction, with minimal immunoreaction in the sarcoplasm (Figure 14b,c). In contrast, the exosome-treated groups (groups III and IV) showed increased immuno-reaction in the cardiac muscle fibers, which was very strong in group III (prophylactic group) when compared with that in group IV (Figure 14d,e).

**Figure 15:**
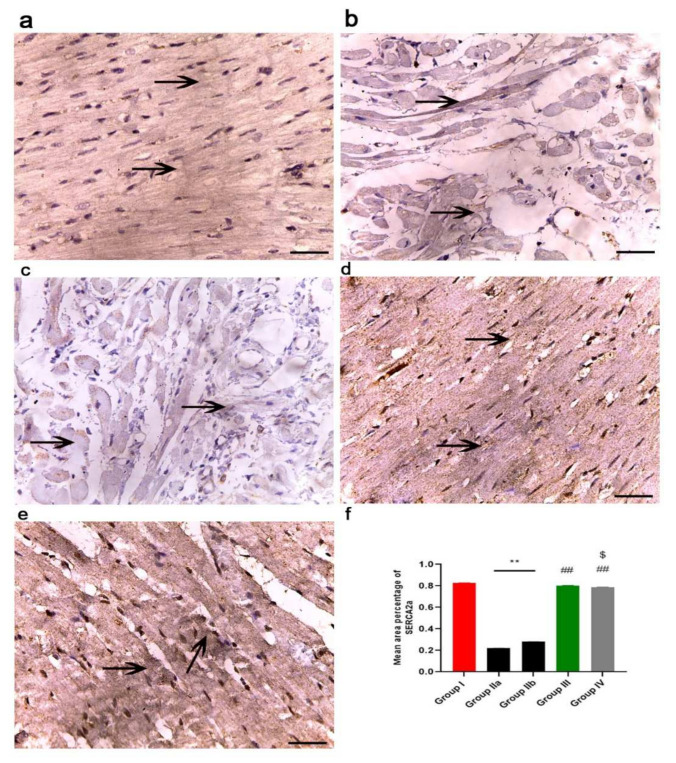
Representative photomicrographs of SERCA2 immuno-stained sections from the left ventricle of the myocardium of the different experimental groups: (**a**) Group I (control group) showed strong immuno-expression of SERCA2 in the myocardium; (**b**) Group IIa (DOX/Trz group) showed minimal immuno-expression of SERCA2 in the myocardium; (**c**) Group IIb (recovery group) showed minimal immuno-expression of SERCA2 in the myocardium; (**d**) Group III (prophylactic group) showed strong immuno-expression of SERCA2 in the myocardium; (**e**) Group IV (curative group) showed moderate immuno-expression of SERCA2 in the myocardium; and (**f**) Histogram representing the mean area percentage of SERCA2 immuno-reaction in all experimental groups. **, significant compared to Group I at *p* ˂ 0.01; ##, significant compared to Group II at *p* ˂ 0.01; $, significant compared to Group III at *p* ˂ 0.05; *n* = 6.

The immunohistochemical reaction of SERCA2 is shown in Figure 15. The sarco(endo)plasmic reticulum Ca^2+^ transport adenosine triphosphatase (SERCA2a) has a central role in modulating Ca^2+^ homeostasis and, therefore, cardiac function. Staining is localized to the sarcoplasm. The control group (group I) showed strong immuno-reaction (Figure 15a), while groups IIa and b (DOX/Trz and recovery groups) showed decreased immuno-reaction, with minimal immunoreaction in the sarcoplasm (Figure 15b,c). In contrast, the exosome-treated groups (groups III and IV) showed increased immuno-reaction in the cardiac muscle fibers, which was very strong in group III (prophylactic group) when compared with that in group IV (Figure 15d,e).

#### 2.11.4. Morphometric Analysis

The mean area percentage of collagen fiber deposition, Cytochrome C, Caspase-3, NCX1, and immuno-expression for all groups are presented in Figure 11f, Figure 12f, Figure 13f, Figure 14f and Figure 15f, respectively. The DOX/Trz groups (group IIa and IIb) showed significant increases (*p* < 0.05) in the mean area percentage of collagen fiber deposition and Cytochrome C and Caspase-3 immuno-expression compared to the control group, while there was significant down-regulation of the immune expression of NCX1 and SERCA2 compared to the control group. Furthermore, the administration of exosomes caused a significant decrease (*p* < 0.05) in the mean area percentage of collagen fiber deposition and Cytochrome C and Caspase-3 immuno- expression compared to DOX/Trz groups and significant up-regulation of the immuno-expression of NCX1 and SERCA2 when compared to the control group. However, the prophylactic group (group III) showed better results compared to group IV in terms of collagen fiber deposition, Cytochrome C, Caspase-3, NCX1, and SERCA2 immuno-expression, which were all near their respective normal levels.

#### 2.11.5. Transmission Electron Microscopy Study

**Figure 16:**
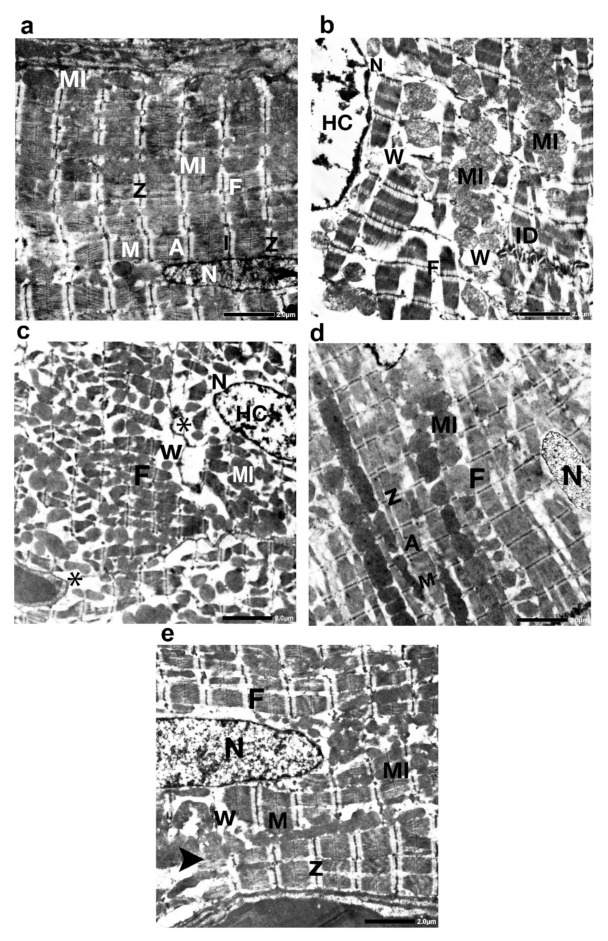
Representative electron micrographs of ultra-thin sections from the left ventricle of the myocardium in the different experimental groups: (**a**) Group I (control group) showed normal sarcomere with well-organized myofibrils (F), a dark band (A), and light bands (I) intersected by Z lines. The A band had an H zone, which was crossed by the M line. A row of mitochondria (Mi) and the nucleus (N) were seen; (**b**) Group IIa (DOX/Trz group) showed disrupted and disorganized myofibrils (F) with areas of fibril loss (arrowhead), distorted mitochondria (Mi), and an irregular nucleus (N) with heterochromatin margination (HC). Wide separation of the fibril bundles (W) with vacuoles (V) and interrupted intercalated disc (ID) were observed; (**c**) Group IIb (recovery group) showed disrupted and disorganized myofibrils (F) with areas of fibril loss (arrowhead), distorted mitochondria (Mi), and irregular nucleus (N) with heterochromatin margination (HC), as well as wide separation of the myofibril bundles (W) with vacuoles and areas of fibrosis (*); (**d**) Group III (prophylactic group) showed sarcomere with organized myofibrils (F), a dark band (A), light bands (I), Z lines, and M line, as well as normal mitochondria (Mi) and part of the nucleus (N). A slight separation of a few myofibrils bundles was noticed (W); and (**e**) Group IV (curative group) showed well-organized myofibrils (F) with transverse striation patterns, Z lines, and M lines. Some myofibrils appeared disrupted with areas of lost fibrils (arrowhead). The mitochondria (Mi) appeared in different sizes and shapes. Separation of some myofibrils bundles (W) was observed. TEM, transmission electron microscopy (×3000); *n* = 4.

Group I (control group) showed closely opposing, branching, striated, and attached end-to-end intercalated discs with a normal sarcomere and well-organized myofibrils having a transverse striation arrangement in the form of alternating dark (A) and light (I) bands intersected by Z lines. The middle of each A band was engaged by the H zone, which was bisected by an M line. Elongated or spherical mitochondria were uniformly distributed in rows between the myofibrils (Figure 16a). Group IIa (the DOX/Trz group) showed marked degeneration and disorganization of ventricular myofibrils and even complete fibrinolysis. A discontinuation of the intercalated discs was observed in many sections, whereas in others, it seemed to be underneath the sarcolemma (intercalated disc avulsion). Some cardiomyocytes displayed mitochondrial degeneration with disrupted cristae. Dilated smooth endoplasmic reticulum with discontinuation of sarcolemma was also seen in some cardiomyocytes. The nucleus seemed irregular, with clumping of the peripheral heterochromatin. Moreover, a separation of the myofibril bundles with vacuoles was detected (Figure 16b). Group IIb (recovery group) displayed similar features as group IIa, but there were more extensive areas of fibrosis between the myofibril (Figure 16c). Groups III and IV (exosome-treated groups) displayed mostly well-organized myofibrils with obvious transverse striation patterns, Z lines, and M lines. Group III (prophylactic group) displayed an ultrastructure of the myocardium similar to that of the control group, except for the slight separation of a few fibril bundles (Figure 16d). However, group IV displayed few disrupted myofibrils, with areas of fibril loss. The mitochondria had different shapes and sizes. Furthermore, a separation of some myofibril bundles was observed (Figure 16e).

## 3. Discussion

The combined use of Trz and DOX has greatly improved the survival of breast cancer patients; however, it is considered a double-edged weapon due to the increased risk of cardiotoxicity [17]. In the present study, we developed a DOX/Trz cardiotoxicity rat model that resembles the protocol used in cancer therapy, concerning the sequential administration of repetitive doses of the two drugs, in order to delineate the pathogenic pathways involved in their induced cardiotoxicity and to assess the cardio-protective mechanisms of systematically infused MSC-derived exosomes.

A combined use of Trz with DOX can augment the cardiomyocyte damage through a ‘dual-hit’ mechanism, which involves the suppression of the survival signaling pathway neuregulin-1 and triggering of angiotensin II/ NADPH oxidase axis, providing the ability to further increase reactive oxygen species production and alter anti-apoptotic signaling pathways in cardiomyocytes [18,19].

In the current study, the cardiotoxicity in group II (DOX/Trz) was confirmed by a significant elevation of serum markers indicating myocardial injury. This was accompanied by a significant decrease in cardiac antioxidant enzyme activities when compared to the control group. Further, echocardiography showed significant cardiac function decline in terms of LVEF and fractional shortening. Moreover, histological data demonstrated marked myocardial degeneration in the form of myofibrillar loss, cytoplasmic vacuolization, Zenker’s necrosis, inflammatory cell infiltration, and fibrosis. Additionally, there were dramatic changes in the ultrastructure of cardiac cells, manifested as loss of myofibrillar normal striation pattern, disarray, vacuolization, irregular folding of the nuclear surface, and nuclear chromatin condensation, which are typical features of apoptosis. Additionally, the mitochondria were condensed with inner wrinkled crista and heterogenous in size and shape. Consistent with these observations, Nicol et al., 2021 [20], and Milano et al., 2019 [21], have demonstrated the cardiac toxicity induced by combined administration of DOX/Trz, in the form of declined left ventricular ejection fraction, augmented cardiac oxidative stress, and cardiac atrophy.

DOX-induced oxidative damage of cellular and mitochondrial membranes results in increased expression of p53 [22,23]. Upon activation, p53 transcriptionally up-regulates the gene expression of the pro-apoptotic members, including Bax and Puma, while down-regulating the gene expression of the anti-apoptotic Bcl-2, thereby activating the intrinsic apoptotic pathway [24]. Puma contributes to p53-mediated apoptosis by indirect induction of mitochondrial outer membrane permeabilization and direct binding with Bcl-2 proteins, preventing their inhibitory effects on Bax. Moreover, DOX binds to topoisomerase IIB (TOP2B) protein and DNA, resulting in double-strand DNA breaks, as well as activating the DNA damage pathway with induction of cardiomyocyte apoptosis [25]. Normally, the cardiomyocytes attempt to repair this damage via the Neuroglin-HER2 pathway but, in the presence of Trz, an anti-HER2 binding agent, the HER2 function would be compromised, resulting in significant damage to cardiomyocytes [26].

In this context, the DOX/Trz group (group II) in the current study developed cardiomyocyte apoptosis, as evidenced by the significant up-regulation of pro-apoptotic genes (e.g., BAX, Puma) and p53, accompanied by significant down-regulation of DNA TOP2B and the anti-apoptotic Bcl2 gene. Moreover, we observed significantly increased immune expression of cytochrome C and Caspase-3 combined with significantly decreased protein expression of HER2. 

Several studies on cardiomyocytes, as well as those in adult hearts, have reported that ErbB2 inhibition itself can result in the dysfunction of mitochondria and trigger apoptotic signaling [27]. The high-affinity binding of Trz to HER2 hinders its dimerization ability with other HER receptors. This, in turn, inhibits cardiac cell survival through phosphoinositide 3 kinase/AKT-, MAPK/ERK 1/2-, and FAK-dependent pathways, leading to the inability of cardiomyocytes to cope with the added stress [28]. Indeed, HER2 blocking has been reported to trigger ROS accumulation and stimulate apoptosis within the cardiomyocytes, ultimately leading to cardiac dysfunction [29]. MAPK are key mediators of the mitochondrial-dependent apoptotic pathway, which are protein kinases of serine/threonine type with three major signaling cascades, identified as: the c-Jun N-terminal kinase (JNK), extracellular signal-related kinases (ERK1/2), and the p38 kinase (p38) [30]. Both JNK and p38 MAPK are activated by ROS, consequently resulting in the induction of apoptosis. Indeed, JNK and p38 can trigger apoptosis through the phosphorylation of specific targets and, hence, activation of downstream apoptotic mediators, such as Bax and p53 [31].

The PI3K/Akt signaling pathway is a crucial cell survival pathway in cardiomyocytes [32]. Akt activation results in the phosphorylation and inactivation of several downstream apoptotic mediators, such as caspase-9 and BAD, thus suppressing the intrinsic pathway of apoptosis [33]. PI3K/Akt signaling is negatively modulated by PTEN, a phosphatase enzyme that accomplishes its function through PI3K dephosphorylation [34]. In this regard, mutual crosstalk has been reported between the p53 and AKT pathways [35]. AKT has been reported to enhance the ubiquitination and subsequent proteosomal degradation of p53, negatively modulating its transcriptional activity [36]. Meanwhile, p53 can induces the gene expression of PTEN, thus suppressing AKT activation [37]. In this context, group II (DOX/Trz) in the current study showed significantly increased PTEN gene expression, accompanied by a significant reduction in cardiac AKT and ERK phosphorylation and marked up-regulation of JNK phosphorylation.

Moreover, group II (DOX/Trz) displayed myofibril loss in cardiomyocytes, as evidenced by the significantly decreased expression of cTnn1, BMHC, and MLC2v genes and significantly decreased left ventricular function when compared to the control group. This is in agreement with a previous study conducted by ElZarrad et al. (2014), in which DOX/Trz significantly induced the down-regulation of cardiomyocyte gene expression with selective proteasome degradation of their proteins, causing a thinning of the left ventricular wall and significantly decreased left ventricular function [38]. DOX/Trz also decreased cardiac troponins in cultured rat neonatal cardiomyocytes, as well as left ventricular tissues of mice [39]. Moreover, DOX/Trz decreased the expression of sarcomeric actin and myosin light chain and heavy chain (MLC 1, MLC 2, BMHC), which are known to play important roles in ventricular adaptation to stressors such as pressure or volume overload. Additionally, MLC2 has been shown to modulate cardiac contractility and trigger troponin-I phosphorylation in response to hemodynamic demands [40].

Furthermore, group II in the current study showed disturbances in cardiomyocyte Ca^2+^ homeostasis, as indicated by a significant decline of SERCA2a gene and protein expression. The sarcoplasmic reticulum Ca^2+^ pump (SERCA2a) plays a pivotal role in intracellular calcium mobilization and, thus, myocardial contractility [41]. The decrease in sarcoplasmic reticulum Ca^2+^ ATPase activity and Ca^2+^ uptake is responsible for the abnormal Ca^2+^ homeostasis in cardiomyocytes of failing hearts [42]. DOX/Trz treatment also affected cardiac NCX1, a sodium/calcium exchanger crucially involved in the maintenance of calcium homeostasis in the heart. This was evident by decreased NCX1 immunostaining in group II cardiac tissue compared to controls. Notably, perturbations in the expression and function of NCX1 have been demonstrated in various clinical and experimental settings of cardiac dysfunction [43], including DOX-induced cardiac toxicity [44,45]. Additionally, Ujihara et al. have reported maintaining NCX1 activity as a promising strategy for protection against heart failure [46].

Therefore, the results and observations of the current study highlighted three pathways—apoptosis, myofibril loss, and calcium homeostasis disorder—involved in DOX/Trz-induced cardiotoxicity. Thus, a therapeutic agent that can affect all these pathways is expected to have great efficacy.

Mesenchymal-stem-derived exosomes have been suggested as a favorable therapeutic strategy, having several advantages in the diseased heart when compared to transplanted stem cells [47]. As shown through functional enrichment analysis, MSC exosome proteins are involved in cell morphogenesis, proliferation, adhesion, and migration. The integration of MSCs differentiation- and self-renewal-related genes and MSC-conditioned media proteomes with the proteomes of MSCs-derived exosomes has revealed (1) MSC-associated antigens, (2) cell adhesion proteins, (3) surface receptors (platelet-derived and epidermal growth factor receptors), and (4) signaling molecules as potential microvesicle protein candidates that may be associated with the therapeutic effects of MSCs-derived exosomes [48]. 

NRG-1 is an epidermal growth factor (EGF)-related protein that signals through ErbB receptors with various regulatory effects in the heart. NRG-1 is synthesized by and released from endothelial cells in the endocardium and myocardial microvasculature in response to mechanical stretching, hypoxia, and signaling peptides [49]. Solubilized NRG-1 binds to adjacent cardiomyocytes by ErbB, leading to the induction of various downstream intracellular signaling pathways involved in the maintenance of cardiac function and structure [50]. Further, NRG-1/HER2 signaling is essential to cardiomyocyte survival, as the binding of NRG-1 initiates cell survival pathways, which inhibit apoptosis and maintains cardiac function [40]. Indeed, NRG-1/HER2 signaling results in activation of the MAPK signaling pathway, leading to the up-regulation of ERK1/2, critical proteins for signaling cell survival. Of note, ERK1/2 have been reported as being able to stabilize myofibril structure, inhibit apoptosis and, thus, stimulate cell survival [51]. Furthermore, NRG-1 signaling activates the PI3K/AKT signal transduction cascade [50]. AKT can alter mitochondrial respiration, thereby decreasing ROS production and increasing cell survival [52]. Additionally, AKT activation triggers endothelial nitric oxide synthase production and alters the expression of Bcl-2 family proteins.

In the current study, MSCs-derived exosomes were intraperitoneally injected into a DOX/Trz-induced cardiotoxicity rat model, either as a protective or therapeutic intervention, in order to elucidate the underlying cardioprotective mechanisms. Interestingly, the treatment groups (groups III and IV) showed significantly decreased serum markers of myocardial injury and a significant improvement in redox homeostasis when compared with group II (DOX/Trz), with better results in group III than group IV. Echocardiography showed a significantly improved cardiac function of LVEF with the enhancement of fractional shortening when compared with group II (DOX/Trz). These results were confirmed histologically, as we observed a marked improvement in DOX/Trz-induced cardiac histopathological changes, with significantly decreased Cytochrome C and Caspase-3 immuno-expression when compared with group II (DOX/Trz). Furthermore, the cardiac ultrastructure was improved to normal or near normal in group III rather than group IV. This cardiac ultrastructure improvement was characterized by highly organized myofibrils with a normal striation pattern, abundant mitochondria with numerous cristae, and a normally appearing rod-shaped nucleus. In agreement with the observations in the current study, Sun et al., 2018 [52], have revealed that MSCs-derived exosomes could alleviate the inflammatory cardiomyopathy caused by DOX cardiotoxicity by improving the inflammatory micro-environment of the myocardium, as well as alleviating the apoptosis of cardiomyocytes through significantly increased LVEF and LVFS, indicating improved cardiac function. 

Next, we investigated whether treatment with MSCs-derived exosomes could restore the key cell survival signaling pathways in cardiomyocytes. Interestingly, we found that injection of MSCs-derived exosomes restored cardiac expression of NRG-1/HER2, MAPK/ERK, and PI3K/AKT, accompanied by down-regulation of PTEN gene expression. Moreover, this treatment suppressed the phosphorylation of cardiac JNK, indicating the implication of these key pathways in the cardio-efficacy of the MSCs-derived exosomes. Furthermore, the treatment groups in the current study showed significant down-regulation of pro-apoptotic genes (BAX, Puma) and p53 protein, accompanied by significant up-regulation of anti-apoptotic Bcl2 gene and tob2p protein when compared to group II (DOX/Trz), again, with better results in group III rather than group IV.

Concomitant with these results, Fukazawa et al. have shown that the apoptotic effect of DOX was significantly decreased when neonatal rat cardiomyocytes were co-cultured with recombinant NRG-1. This NRG-1 effect was associated with the activation of AKT. In contrast, over-expression of a dominant-negative AKT by adenoviral infection prevented this anti-apoptotic NRG-1 effect, suggesting that AKT is necessary for the anti-apoptotic effects of NRG-1 [53]. Another study by Rohrbach et al. has also reported the inhibition of DOX-induced cytochrome c release and caspase-3 activation by NRG-1. Additionally, NRG-1 also inhibited DOX-induced imbalance in the ratio of Bcl-xS to Bcl-xL [54]. Interestingly, Figliolini et al. have recently shown that ADSCs-derived extracellular vesicles protected against muscle inflammation and damage through an NRG-1-dependent mechanism [55]. Wen et al. have demonstrated that MSCs-derived exosomes inhibit hypoxia-induced cardiomyocyte apoptosis by targeting the PTEN/AKT pathway [56]. Similarly, Sun et al. have reported a protective effect of bone marrow stromal cell-derived exosomes against cardiomyocyte apoptosis and myocardial ischemia-reperfusion injury through the suppression of PTEN expression and activation of the PI3K/AKT signaling pathway [57]. Furthermore, MSCs-derived exosomes transplantation in MI-heart has reduced infarct size and restored cardiac contractile function. These cardio-protective effects were attributed to reduced PTEN expression and secondary activation of the AKT and ERK signaling pathways in cardiac tissues [58].

Furthermore, we investigated whether treatment with MSC-derived exosomes could restore cardiac myofibril through the NRG-1/ErbB signaling pathway. Interestingly, we found that the exosome treatment groups showed significantly increased gene expression of NRG-1, cTnn1, BMHC, and MLC2v when compared with group II (DOX/Trz), with better results in group III rather than group IV. These results were confirmed histologically through improvement of the ultrastructure of cardiomyocytes. Concomitant with our results, Pentassuglia and Sawyer have stated that recombinant NRG-1 reduced DOX/Trz-induced myofibril damage by activating both PI3K and MAPK pathways in cardiomyocytes, while ErbB2 inhibition prevented the activation of these pathways by NRG-1. In addition, MAPK inhibition—but not PI3K—prompted myofilament injury in adult rat cardiomyocytes, similar to that observed in cardiomyocytes treated with an ErbB2 antibody or inhibitor. These results suggest that NRG-1-mediated myofibril protection requires ErbB2 and MAPK activation [27]. Further, NRG-1/ErbB signaling has been reported to attenuate the DOX/Trz-induced cardiac troponin protein loss both in mouse hearts and in cultured neonatal rat cardiomyocytes. NRG-1 prevented the down-regulation of cTnI and cTnT proteins in cultured cardiomyocytes by increasing transcription and translation while decreasing the degradation of these proteins [39]. NRG-1 also increases GATA4 phosphorylation. GATA4 is a transcriptional factor that plays a crucial role in cardiac development through regulating genes that encode cardiac sarcomeric proteins. Accordingly, NRG-1-mediated GATA4 phosphorylation was suppressed through inhibition of the ErbB2 receptor or the MAPK pathway [40]. 

Kim et al. have analyzed MSCs-derived exosome proteomics and revealed that the MSC-MV proteome included five positives and two variable known markers of MSCs, as well as 43 surface receptors and signaling molecules controlling the self-renewal and differentiation of MSCs. MSC-MV proteome analysis revealed potential protein candidates that may be associated with the therapeutic effects of MSC-MVs: (1) Surface receptors (PDGFRB, EGFR, and PLAUR), (2) signaling molecules (RRAS/NRAS, MAPK1, GNA13/GNG12, CDC42, and VAV2), (3) cell adhesion molecules (FN1, EZR, IQGAP1, CD47, integrins, and LGALS1/LGALS3), and (4) MSC-associated antigens (CD9, CD63, CD81, CD109, CD151, CD248, and CD276) [59]. Therefore, the MSC-MV proteome provides a comprehensive basis for understanding the potential of MSC-MVs to affect tissue repair and regeneration.

Therefore, the cardio-protective characteristics of MSCs-derived exosomes could be accredited to the presence of EGFR on the surface of exosomes. NRG-1 acts with EGFR by the NRGs/EGFLs:p-ERBB4cyt2:p-6Y-ERBB2 pathway (http://www.reactome.org/, accessed date 25 December 2021). Another explanation for this activity could be attributed to the recognition of active CD73 as a surface marker of MSCs-derived exosomes (data available at www.exocarta.org, accessed date 25 December 2021) [60]. CD73 is the major enzyme responsible for the formation of extracellular adenosine from released adenine nucleotides [61]. The presence of biologically active CD73 on exosomes triggers adenosine receptors and induces adenosine-induced phosphorylation of ERK and AKT, providing another mechanistic basis for the paracrine effect of MSCs in enhancing cardiac tissue repair and reducing cardiac tissue injury.

On the other hand, there is growing evidence that MCSs-exosomal miRs, including miR-19a, mirR-21, miR-26a, miR-210, miR-23a-3p, and miR-146a, participate in the cardioprotective effects of MSCs. Exosomal miR-21 restrains PTEN and activates the PI3K/Akt pathway. PTEN has been documented as one of miR-21’s target genes. Indeed, miR-21 inhibits apoptosis and promotes cell proliferation through PTEN-dependent PI3K/Akt activation [62]. Interestingly, the gain of function of miR-21 can efficiently reduce ischemia perfusion injury through the down-regulation of PTEN expression [63]. Furthermore, a recent study by Wang et al. has confirmed the role of miR-26a in the regulation of PI3K/Akt and JAK/STAT signal transduction pathways through suppressing the up-regulation of PTEN [64].

The exosome-treated groups in the current study also showed maintained calcium homeostasis, as evidenced by restored SERCA2a gene and protein expression, as well as NCX1 protein expression. In this line, Timolati et al. (2006) have demonstrated that NRG-1 treatment increased SERCA proteins, leading to an increase in calcium amplitude and fractional shortening in DOX-treated cultured cardiomyocytes [65].

## 4. Materials and Methods

### 4.1. Experimental Animals

Eight-week-old adult male albino rats weighing 250–270 g were obtained from the Experimental Animal Unit of the Faculty of Veterinary Medicine, Benha University, Egypt. Rats were housed in clean cages and allowed free access to a standard rodent diet and clean tap water. Animals were allowed to adapt to the lab environment, which was controlled in terms of room temperature (23 ± 3 °C) and light (12 h cycle starting at 8:00 a.m.). All experimental procedures were approved by the institutional review board for animal experiments of the Faculty of Medicine, Benha University, Egypt. This study was carried out in strict adherence to the recommendations in the Guide for the Care and Use of Laboratory Animals of the National Institutes of Health (NIH publication 85–23, revised 2011).

### 4.2. Preparation of MSCs-Derived Exosomes

The supernatant of MSCs, representing conditioned media, was used for the preparation of MSCs-derived exosomes. First, rat-adipose-derived MSCs (ADMSCs) were prepared in the Central Lab of the Faculty of Medicine, Benha University [66,67]. The MSCs were cultured overnight in fetal bovine free Dulbecco’s Modified Eagle Medium (DMEM), to which 0.5% human serum albumin (HSA) (Sigma-Aldrich, St. Louis, MO, USA) was added. The rationale behind this step was to prevent cell death and decrease the number of apoptotic bodies and cell debris that might be released into the conditioned media [68]. Trypan blue exclusion was used to check the viability of the cells cultured overnight, which revealed more than 99% viability. Thereafter, cells were plated for 7 days at 4000 cells/cm^2^. Cells were trypsinized, counted, and re-incubated at a density of 2000 cells/cm^2^ in expansion medium on day 7 for another 7 days (end of passage 1). The expansion was repeated until the third passage. This was followed by conditioned medium collection and storage at −80 °C. To isolate the exosomes, the medium was centrifuged to remove debris at 2000× *g* for 20 min and then ultracentrifuged for 1 h at 100,000× *g* in an SW41 swing rotor at 4 °C (Beckman Coulter, Fullerton, CA, USA). The isolated exosomes were further washed with serum-free M199 (Sigma-Aldrich) pre-mixed with 25 mM of 4-(2-hydroxyethyl)-1-piperazineethanesulfonic acid (HEPES; pH = 7.4) and subjected to a second ultracentrifugation in the same conditions. The collected exosomes were then stored at −80 °C until further needed.

### 4.3. Characterization of MSC-Derived Exosomes

The exosomes were isolated from the supernatant of MSCs cultured in FBS-deprived DMEM (first, second, and third passages). The isolated exosomes were kept in 2.5% glutaraldehyde in HSA for 2 h for fixation. Following a washing step, the exosomes were ultracentrifuged and suspended in HSA (100 μL). Then, for observation by transmission electron microscopy (Hitachi H-7650, Hitachi, Tokyo, Japan), 20 μL of exosomes was loaded onto a formvar/carbon-coated grid, negatively stained with 3% aqueous phosphotungstic acid for 1 min [69], and the Bradford method (BioRad, Hercules, CA, USA) was used to quantify the protein content of exosomes. Additionally, PKH-26 (Sigma-Aldrich, St. Louis, MO, USA) was used to confirm exosomal localization within the cardiac tissue. Briefly, the exosome pellet was diluted to 1 mL using the PKH-26 kit solution. Then, fluorochrome (2 μL) was added to exosome suspension and incubated for 15 min at 38.5 °C. After that, the exosome suspension was mixed with serum-free HG-DMEM (7 mL) and ultracentrifuged at 100,000× *g* at 4 °C for 1 h. The final pellet was rapidly resuspended in HG-DMEM and then stored at −80 °C until further use in the experimental animals [70].

### 4.4. Western Blot for Characterization and Localization of Exosomes

For exosome identification, we used CD63, CD81, and CD83, while for exosome localization in cardiac tissues, we used CD29, CD44, and CD 73. The antibodies used were antigen-affinity-purified rabbit monoclonal [EPR4244] to CD81 (1/1000–1/10,000 dilution, ab109201, Abcam, Cambridge, UK), rabbit polyclonal antibody against CD63 (1/1000 dilution, A5271, ABclonal), rabbit polyclonal antibodies against CD83 (1:1000 dilution, PA5-96044, ThermoFisher Scientific, Waltham, MA, USA), rabbit monoclonal [EPR16895] against CD29 (1/2000 dilution, ab179471, Abcam, Cambridge, UK), rabbit monoclonal antibody [EPR18668] against CD44 (1/1000 dilution, ab189524, Abcam, Cambridge, UK), and rabbit polyclonal antibody against CD73 (1:500 dilutions, PA5-81614, ThermoFisher Scientific, Waltham, MA, USA). RIPA buffer was used for the isolation of proteins from the isolated exosomes. A total of 20 ng of protein was loaded onto 4–20% polyacrylamide gradient gels and separated by SDS-PAGE. The separated protein was blotted onto a PVDF membrane, which was further blocked by incubation for 1 h in 5% non-fat dry milk, Tris-HCl, and 0.1% Tween 20. Primary antibodies (CD81, CD63, CD83, CD29, CD44, and CD73 antibodies) were added to membranes and incubated at 4 °C overnight. β-actin was used as a loading control. The membrane was then washed twice with 1% TBST, and appropriate secondary antibodies were then added and incubated for 2 h at room temperature. Another washing step was applied by washing the membrane twice with 1% TBST. The intensity of immunoreactivity was analyzed by densitometry, in order to quantify the amounts of CD81, CD63, CD83, CD29, CD44, and CD73, using β-actin for total protein normalization. The images were analyzed using software on the ChemiDoc MP imaging system (version 3) produced by BioRad (Hercules, CA, USA).

### 4.5. Direct Labeling of MSCs with Iron Oxide

MSCs were marked using 50 μm iron oxide in 4 mL of RPMI media for 30 min, followed by centrifugation at 2000 rpm for 10 min to separate the iron-labeled MSCs [71]. All labeled MSCs released exosomes that contained iron oxide [72] as, after the cells were directly labeled, the labeled exosomes were created inside the cells and released. One of the advantages of the direct labeling method is that it is simple and relatively physiological, without any need for gene modification.

### 4.6. Prussian Blue Staining

The Prussian blue reaction involves the treatment of sections with acid solutions of ferrocyanides. Any ferric ion (+3) present in the tissue combines with ferrocyanide, resulting in the formation of a bright blue pigment called ferric ferrocyanide (or Prussian blue). Iron-labeled MSCs injected were detected by Prussian blue staining in the cardiac tissues, where eosin was used as a counterstain [73].

### 4.7. Experimental Chemicals

− Doxorubicin hydrochloride (DOX), as a powder, was purchased from Genentech, Inc., South San Francisco, CA, USA [Product code; D1515- ID 24893465].
− Dosage of DOX: Doxorubicin hydrochloride (DOX) powder was dissolved in sterile distilled water before administration. Each 80 mg of DOX powder was dissolved in 10 mL of sterile distilled water, yielding a multi-dose solution containing 8 mg/mL. Each rat of the tested groups was injected intraperitoneally with 0.13 mL of solution (1 mg/dose), according to Nicol et al. [20].
− Trastuzumab (Trz), as HERCEPTIN^®^ lyophilized powder (440 mg), was purchased from Genentech, Inc., South San Francisco, CA, USA [Product code; SAP-10086840].
− Dosage of Trz: Trastuzumab (Herceptin) lyophilized powder was dissolved in sterile distilled water before administration. Each vial (440 mg) of Herceptin was dissolved in 60 mL of sterile distilled water to yield a multi-dose solution containing 7.3 mg/mL. Each rat of the tested groups was injected intraperitoneally with 0.12 mL of solution (0.87 mg/dose), according to Nicol et al. [20].
− Sterile distilled water was purchased from ATECO Pharma (Kaliobeya, Egypt).
− All drugs and chemicals were of the highest purity and analytical grade and were freshly prepared before each experiment.

### 4.8. Experimental Design

**Figure 17:**
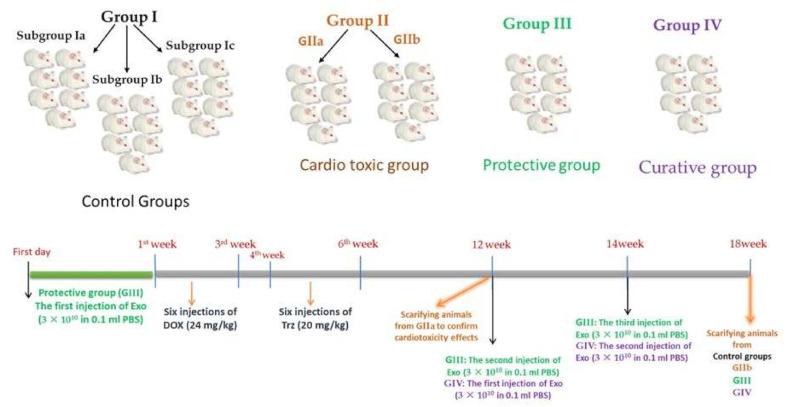
Timeline of the experimental design.

Forty-nine male rats were randomly allocated into four groups, as follows (Figure 17):

**Group I (control group; *n* = 21):** Rats were fed a regular chow diet all the time during the experiment. The rats were divided equally into three sub-groups, each having seven rats:

**Sub-group Ia:** Rats were left without intervention to measure the basic parameters.

**Sub-group Ib:** Rats were injected with sterile distilled water as a vehicle—injected intraperitoneally (i.p.)—in the same dosing regimen as DOX/Trz treatments.

**Sub-group Ic:** Rats were i.p. injected with 0.2 mL phosphate-buffered saline (PBS) in the same regimen as exosomes administration (vehicle for exosomes).

**Group II: (Cardiotoxic group; *n* = 14):** DOX was administered in six i.p. injections over two weeks (total cumulative dose: 24 mg/kg). Then, after one week, Trz was administered in six i.p. injections over two weeks (total cumulative dose: 20 mg/kg). The rats were then divided equally into two sub-groups:

**Sub-group IIa:** Rats were sacrificed six weeks after the final Trz injection to study the cardiotoxicity effects due to DOX/Trz administration by histopathology, immunohistochemistry, and transmission electron microscopy.

**Sub-group IIb:** Rats were sacrificed at the end of the experiment to study the recovery from the DOX/Trz cardiotoxicity effects.

**Group III:** (Protective group; *n* = 7): One dose of exosomes (3 × 10^10^), re-suspended in 0.1 mL PBS, was intraperitoneally (i.p.) injected one week before the induction of cardiotoxicity. Then, six weeks after the final Trz injection, two doses of exosomes (3 × 10^10^), re-suspended in 0.1 mL PBS, were intraperitoneally (i.p.) injected two weeks apart. The rats were sacrificed four weeks after the last exosome injection [21,74].

**Group IV:** (curative group; *n* = 7): Six weeks after the final Trz injection, two doses of exosomes (3 × 10^10^), re-suspended in 0.1 mL PBS, were intraperitoneally (i.p.) injected two weeks apart. Rats were sacrificed four weeks after the last exosomes injection [21,74].

### 4.9. Sampling

At the end of the experimental procedure, all rats were anesthetized with ketamine– xylazine (K, 100 mg/kg; X, 10 mg/kg) by intramuscular injection. The rats were fixed on an operating table, and blood samples were obtained from the retro-orbital venous plexus using a fine-walled Pasteur pipette. This was followed by vascular perfusion fixation using 10% buffered formol saline through the left ventricle. Following fixation, the hearts of rats of all groups were dissected for both histopathological examination—by Hematoxylin and Eosin (H&E), Masson’s trichrome, electron microscopy, and immunohistochemical staining for Cytochrome C and Caspase-3—in addition to biochemical testing and molecular analysis by Real-Time PCR, Western blot, and spectrophotometry.

### 4.10. Determination of Serum Cardiac Enzymes and Cardiac Tissue Oxidative Stress Indices

Orbital blood was collected under anesthetized conditions using ketamine–xylazine (K, 100 mg/kg; X, 10 mg/kg) by intramuscular injection. Blood samples were centrifuged at 4 °C to separate the serum, which was then stored at −80 °C until assay. The serum levels of troponin-1 (Cat No: 240 001), creatine phosphokinase–MB isoenzyme (CK–MB) (Cat No: 239 001), and serum lactate dehydrogenase (LDH) (Cat No: 279 001) were measured using commercial kits supplied by Spectrum diagnostics (Cairo, Egypt) and were read on a spectrophotometer following the manufacturer’s instructions. LDH assay was carried out, based on its ability to catalyze the reaction between NADH and pyruvate to produce L-Lactate and NAD. The activity was determined by measuring the decrease in absorbance at 340 nm by the kinetic method. CK-MB activity was determined based on the rate of NADPH formation utilizing hexokinase and glucose-6-phosphate dehydrogenase-catalyzed reactions. The absorbance was measured at 340 nm. Troponin I concentration was determined using the latex enhanced immunoturbidimetric method. The absorbance was measured at 505 nm. Measurements were performed in technical triplicates, and the levels are presented as ng/mL or U/L.

For the assessment of cardiac oxidative stress, heart tissue homogenates were prepared in saline, which were then used for the evaluation of oxidative stress indices. The levels of reduced glutathione (GSH) and malondialdehyde (MDA) and the activity of superoxide dismutase (SOD) in heart homogenate were tested using commercially available kits (Biodiagnostics, Giza, Egypt), following the protocol provided by the manufacturer. The enzyme activities are expressed as U/mg protein. Protein was determined according to the method of Lowry et al. [75].

### 4.11. Echocardiography

At the end of the experimental period, echocardiography was performed in rats using a MyLab^TM^ 30 VET Gold (Esoate Co., Florence, Italy), equipped with a high-frequency 4–8 MHz phased array transducer, under ketamine–xylazine (K, 100 mg/kg; X, 10 mg/kg; by intramuscular injection). The thoracic walls of the rats were shaved clean, after which they were placed in the proper posture (semi-left lateral position with upright tilt). Ultrasound gel was placed on the thorax to optimize visibility. Doppler, two dimensional (2D) guided M-mode images were recorded from parasternal long-axis, parasternal short-axis, and apical four-chamber views. The left ventricular end-systolic and diastolic diameters (LVSD and LVDD) were estimated [76]. Then, fractional shortening (FS) and ejection fraction (EF) were calculated using the following formulae:

FS % = (LVDD – LVSD)/LVDD × 100

EF % = (LVDD^3^ – LVSD^3^)/LVDD^3^ × 100

All parameters depended on the mean values of three cardiac cycles [77].

### 4.12. Gene Expression Profile

For RNA extraction from the cardiac tissues, TRIzol (Invitrogen) was used, following the manufacturer’s instructions. The extracted RNA was tested for purity, and RNA concentration was assayed using a Nano-Drop 2000C spectrophotometer (Thermo Scientific, Waltham, MA, USA). RNA purity for all samples was >1.9 at an absorbance ratio A260/A280. The integrity of RNA was then verified by loading onto 2% agarose gel using a gel electrophoresis image (Gel Doc. BioRad, Hercules, CA, USA), according to Ref [78]. RNA was used for complementary DNA (cDNA) synthesis using SensiFast cDNA synthesis kits (Sigma Bioline, London, UK), according to the manufacturer’s instructions.

Quantitative PCR was performed, according to Ref [78], using Maxima SYBR Green/ROX qPCR master mix (2×) (Thermo Scientific, Waltham, MA, USA). Each PCR reaction consisted of 0.3 μmol L^−1^ of each forward and reverse primer, 10 nmol L^−1^/100 Nm ROX Solution, 500 ng per reaction of cDNA (except for NTC and cDNA control), and 12.5 μL Maxima SYBR Green qPCR Master Mix (Maxima SYBR Green qPCR, ThermoFisher Scientific). The mix was completed to a final volume of 25 μL with nuclease-free water. Primer pairs for selected target and reference genes (NRG-1, cTnn1, BMHC, MLC2v, BAX, Bcl-2, puma, CTGF, collagen1a1, MMP9, SERCA2a, PTEN, iNOS, and GAPDH) were purchased from Genwez (South Plainfield, NJ, USA); see Table 2. The reaction was completed using a two-step protocol AriaMx Real-Time PCR (Agilent Technologies, Santa Clara, CA, USA), as follows: initial denaturation for 10 min at 95 °C, then 40 cycles (15 s each) of denaturation at 95 °C, followed by annealing/extension for 60 s at 60 °C. Following PCR, a melting curve protocol was performed by heating for 30 s at 95 °C, then for 30 s at 65 °C, and 30 s at 95 °C. GAPDH was used as a housekeeping gene for normalization of the expression levels of target genes.

For miRNA analysis, total miRNA was extracted using an miRNeasy kit (Qiagen). Taqman methods were applied to assess miRNA expression. Reverse transcriptase reaction was carried out using a Taqman microRNA Reverse Transcription Kit and Taqman microRNA assay stem-loop primers (Applied Biosystems). Taqman microRNA assay and Taqman universal PCR master mix reagents (Applied Biosystems) were used for quantitative real-time analysis, and all procedures were performed following the instructions in the manufacturer’s kits. The relative miRNA levels were normalized to U6 as an internal reference for miRNA in tissues, and to two spikes in miRNAs—cel-miR-39 and cel-miR-238 (Applied Biosystems)—as reference for miRNA in the exosomes. The formula RQ = 2^−ΔΔCt^ was used to calculate relative gene expression ratios (RQ) of mRNA and miRNA between the treatment and control groups [94].

### 4.13. Enzyme-Linked Immunosorbent Assay (ELISA) 

Heart tissue homogenate was used to assess the protein levels of P53 (Invitrogen, ERA47RB), top2b (HUFI01088, Ireland), ErbB2 (LSBio, Seattle, WA, USA, LS-F11388), Erb4 (Wuhan Fine Biotech, Wuhan, China), AKT (DEIA1881, Creative Diagnostics, Shirley, NY, USA), Phospho-AKT (MBS9511022, MyBiosource, San Diego, CA, USA), JNK (MBS2508439, MyBiosource, San Diego, CA, USA), Phospho-JNK (PEL-JNK-T183, RayBiotech, GA, USA), ERK (MBS034880, MyBiosource, San Diego, CA, USA), Phospho-ERK (DYC1825-2, R&D Systems, Minneapolis, MN, USA), and Phospho-STAT3/STAT3 (ab176666, Abcam, MA, USA) using the ELISA method and following the manufacturer’s instructions.

### 4.14. Histopathological Analysis

Cardiac tissues were excised and fixed in buffered formol saline (10%). Fixed tissue specimens were used for the preparation of 4–6 µm thick paraffin sections, which were mounted on glass slides for hematoxylin and eosin (H&E) and Masson’s trichrome stains, and positively charged slides were prepared for immunohistochemical (IHC) staining.

For H&E and Masson’s trichrome staining, fixed sections were dehydrated with successive concentrations of ethanol and washed twice in distilled water, followed by staining with H&E and Masson’s trichrome stains. Then, the histological sections were observed, analyzed, and imaged by two blinded experienced investigators using a light microscope (Leica DMR 3000; Leica Microsystem, Wetzlar, Germany) [95].

### 4.15. Immunohistochemical Analysis

Paraffin sections were deparaffinized and hydrated. After blocking the endogenous activity of peroxidase using 10% hydrogen peroxide, the sections were blocked for non-specific reactions and incubated with primary rabbit polyclonal antibodies against Cytochrome c (SAB4502234; 1:50–1:100 dilutions; Sigma-Aldrich, St Louis, MO, USA), rabbit monoclonal antibodies against caspase-3 (ab32351; 1/25–1/100 dilutions; Abcam, Cambridge, UK), mouse monoclonal [C2C12] against NCX1 (ab2869; 5 µg/mL concentration; heat-mediated antigen retrieval performed with citrate buffer of pH 6 before commencing with IHC staining protocol; Abcam, Cambridge, UK), and rabbit polyclonal to SERCA2 ATPase (ab3625; 1 µg/mL concentration; heat-mediated antigen retrieval performed before commencing IHC staining protocol; Abcam, Cambridge, UK). Then, after washing with phosphate buffer, a biotinylated goat anti-rabbit secondary antibody was applied. For localization of the immune reaction, the slides were incubated with labeled avidin-biotin peroxidase, which binds to the biotin on the secondary antibody. Diaminobenzedine was used as chromogen for visualization of the site of antibody binding, which is converted into a brown precipitate by peroxidase [96].

### 4.16. Morphometric Study

The Image-Pro Plus software version 6.0 (Media Cybernetics Inc., Bethesda, MD, USA) was used for calculation of the mean area percentage of collagen fiber deposition, Caspase-3, cytochrome C, NCX1, and SERCA2 immuno-expression. The mean area percentage for each marker was quantified using five images from five non-overlapping fields of each rat in each group.

### 4.17. Transmission Electron Microscopy Study

Glutaraldehyde (1%) was used for vascular perfusion fixation through the left ventricle. Rat hearts were then dissected and incubated in 0.1 M phosphate-buffered solution (PBS; pH 7.4) for 2 h at 4 °C, then washed three times with PBS (10 min each). Samples were post-fixed for 30 min in 1% osmic acid, then washed with PBS three times (10 min each). Fixed specimens were dehydrated using an ascending series of ethyl alcohol (30, 50, 70, 90%, and absolute alcohol) for 30 min at each concentration. Dehydrated specimens were infiltrated for 1 h with acetone and then embedded in Araldite 502 resin. A Leica UCT ultramicrotome was used to cut the plastic molds into semi-thin sections, which were stained with 1% toluidine blue. Following semi-thin section examination, ultra-thin sections were prepared (50–60 nm thick), treated with uranyl acetate for staining, and then with lead citrate for counter-staining. The sections were further analyzed and photographed using an electron microscope (JEOL-JEM-100 SX, Akishima, Japan) at the electron microscope unit of Tanta University (10948426).

### 4.18. Statistical Analysis

The statistical software package SPSS for Windows (Version 16.0; SPSS Inc., Chicago, IL, USA) was used to perform statistical analyses. Differences between various experimental groups were evaluated using one-way analysis of variance (ANOVA; F) and the Kruskal–Wallis test (χ^2^) for parametric and non-parametric data, respectively. Post hoc analysis was then performed to detect differences in pairs. Parametric data are expressed as the mean ± standard error (SEM), non-parametric data are expressed as the median (maximum and minimum), and a *p*-value of <0.05 was considered significant.

## 5. Conclusions

Collectively, the results of the current study demonstrated the preventive and therapeutic potential of MSCs-derived exosomes in DOX/Trz-induced cardiotoxicity through modulation of the cardiac NRG-1/HER2, ERK, JNK, and PI3K/AKT signaling pathways, leading to a superior preventive effect. These findings suggest the MSCs-derived exosomes may serve as potential candidates for adjunctive therapy in cancer patients treated with the DOX/Trz combination.

## Figures and Tables

**Table 1:** Echocardiography results for different study groups.

Group	LVDD (cm)	LVSD (cm)	EF%	FS%
I	7.2	3.78	85.77	47.77
II	7.7 ^a^	5.43 ^a^	65.92 ^a^	30.33 ^a^
III	7.2 ^b^	3.8 ^b,c^	85.28 ^b,c^	47.27 ^b,c^
IV	7.3 ^b^	4.23 ^a,b^	80.83 ^a,b^	42.18 ^a,b^

Values expressed as means ± SEM, *n* = 6. LVDD, left ventricular diastolic dimension; LVSD, left ventricular systolic dimension; EF, ejection fraction; FS, fractional shortening. Values with *p* < 0.05 were considered significant. One-way analysis of variance (ANOVA) and post hoc multiple comparisons tests were used. ^a^ *p* < 0.05 compared with Group I (control group); ^b^ *p* < 0.05 compared with Group II (cardio toxic group); ^c^ *p* < 0.05 compared with Group IV (curative group).

**Table 2:** Sequences of primers used for RT-PCR in this study.

Target Genes	Primer Sequence 5’ to 3’	References
Nrg-1	5′-AGCAGACACCAGCTTCAGAC-3′ 5′-CAAGAAGGCAGGGGACCAAA-3′	[79]
cTnnc1	5′-AGCCTGTCCTGTGAGCTGTC-3′ 5′-GGCAGCCTTGAACTCATTCT-3′	[80]
BMHC	5′-GCCAACACCAACCTGTCCAAGTTC-3′ 5′-TGCAAAGGCTCCAGGTCTGAGGGC-3′	[59]
Mlc2v	5′-ACTATGTCCGGGAGATGCTG-3′ 5′-TGGGTAATGATGTGGACCAA-3′	[81]
Bax	5′-GATCAGCTCGGGCACTTTA-3 5′-TGTTTGCTGATGGCAA CTTC-3′	[82]
Bcl-2	5′-AGGAT TGTGG CCTTC TTTGA GT-3′ 5′-GCCG GTTC AGG TACT CAGT CAT-3′	[83]
Puma	5′- GTG TGG AGG AGGAGG AGT GG-3′ 5′-TCG GTG TCG ATG TTG CTC TT-3′	[81]
CTGF	5’-CCTGGTCCAGACCACAGAGT-3′ 5’-TTTTCCTCCAGGTCAGCTTC-3′	[84]
Col1a1	5′-CCTCAGGGTATTGCTGGACAAC-3′ 5′-CAGAAGGACCTTGTTTGCCAGG-3′	[85]
Mmp9	5′-GCTGACTACGATAAGGACGGCA-3′ 5′-TAGTGGTGCAGGCAGAGTAGGA-3′	[86]
SERCA2a	5′-CTCCATCTGCTTGTCCAT-3′ 5′-GCGGTTACTCCAGTATTG-3′	[87]
PTEN	5′-CAATGTTCAGTGGCGGAACTT-3′ 5′-GGCAATGGCTGAGGGAACT-3′	[88]
iNOS	5′-ATGGACCAGTATAAGGCAAGC-3′ 5′- GCTCTGGATGAGCCTATATTG-3′	[89]
GAPDH	5′-AGT TCA ACG GCA CAG TCA A -3′ 5′-TAC TCA GCA CCA GCA TCA CC -3′	[90]
miR-26a	5′-UUCAAGUAAUCCAGGAUAGGCU-3′ 5′-AGCCUAUCCUGGAUUACUUGAA-3′	[91]
miR-21	5′-CGCGCTAGCTTATCAGACTGA-3′ 5′-CGGCCCAGTGTTCAGACTAC-3′	[92]
U6	5′-CTCGCTTCGGCAGCACA-3′ 5′-AACGCTTCACGAATTTGCGT-3′	[93]

## Data Availability

The data that support the findings of this study are available from the authors upon reasonable request from the corresponding author.
